# Comprehensive modelling of coastal landuse and land cover (LULC) dynamics and future changes using random forest, GEE, and MLP–Markov chain integration: a geo-computational approach

**DOI:** 10.1038/s41598-026-60792-4

**Published:** 2026-07-17

**Authors:** P. S. Fathimathu Sahala, S. Kaliraj, Reji Srinivas, Ramesh Madipally, Girish Gopinath, Suresh Devaraj, Parthiban Loganathan

**Affiliations:** 1 National Centre for Earth Science Studies (NCESS), Ministry of Earth Sciences, Govt of India, Thiruvananthapuram, 695011 India; 2 NCESS Ph.D. Scholar, School of Marine Sciences, CUSAT, Kochi, Kerala India; 3 Kerala University of Fisheries and Ocean Studies, Cochin, Kerala 682506 India; 4 Centre for Remote Sensing and Geoinformatics, Sathyabama Institute of Science and Technology, Chennai, Tamil Nadu 600119 India; 5 Department of Engineering Mechanics, KTH Royal Institute of Technology, 11428 Stockholm, Sweden

**Keywords:** Land change modeller, Classification and regression trees (CART), Google earth engine, LULC dynamics, Multi-layer perceptron–Markov chain, TerrSet, Kerala coastal stretch, Climate sciences, Ecology, Ecology, Environmental sciences, Natural hazards

## Abstract

Coastal landuse / land cover (LULC) faces dynamic transformations driven by natural and human activities. This study investigates decadal LULC dynamics and their future projections (2034, 2044, and 2054) along the Ernakulam–Alappuzha coastal stretch of Kerala, India, using Google Earth Engine (GEE)- based time-series Landsat ETM + and OLI images from 2004, 2015, and 2024. GIS-based machine learning (ML) techniques, including Random Forests (RF), Support Vector Machines (SVM), and Classification and Regression Trees (CART), were used to extract LULC features. The spectral indices, such as NDVI (Normalized Difference Vegetation Index), SAVI (Soil-Adjusted Vegetation Index), MNDWI (Modified Normalized Difference Water Index), NDBI (Normalized Difference Built-up Index), NDBaI (Normalized Difference Bareness Index), BSI (Bare Soil Index), and UI (Urban Index), were incorporated to enhance the future LULC trends. Among all ML algorithms, the RF achieved the highest classification accuracy with 95.46%, 92.38%, and 93.94% for 2004, 2015, and 2024, respectively. The LULC patterns indicate pronounced urban expansion, with built-up areas increasing from 109.93 km^2^ (6.47%) in 2004 to 373.49 km^2^ (21.98%) in 2024, largely replacing plantations, which declined from 648.94 km^2^ (38.17%) to 397.03 km^2^ (23.36%). The results reflect an accelerating urbanization trend and a gradual decline of natural and semi-natural coastal land covers. Future LULC projections for 2034, 2044, and 2054, simulated using the Multi-Layer Perceptron–Markov Chain (MLP–MC) model in the Land Change Modeller (LCM) of TerrSet v.20, reveal significant transformations in the Ernakulam–Alappuzha coastal stretch. Validation of the 2024 predicted map against the actual LULC map produced a Kappa value of 0.71, confirming the model’s reliability. The projections indicate a dominant expansion of built-up areas, from 373.49 km^2^ (21.98%) in 2024 to 747.52 km^2^ (43.99%) by 2054, with the most rapid increase occurring between 2024 and 2034 (147.58 km^2^, 39.51%, 14.76 km^2^/year). The changes of LULC reflect the trend of future patterns which accelerate urban growth, primarily through the conversion of plantations, fallow land, vegetation, and wetlands. In contrast, natural and semi-natural land covers are expected to decline markedly, with the steepest changes in the first decade (2024–2034) and slower, continued transformations thereafter. These patterns suggest ongoing urban dominance and degradation of coastal ecosystems, underscoring the need for strategic land-use planning and sustainable coastal management practices.

## Introduction

Land is a fundamental natural resource that sustains human life and economic activities^1^. Over recent decades, natural processes and human interventions have increasingly transformed the Earth’s surface, leading to significant landuse and land cover (LULC) changes driven by environmental and socio-economic pressures^2^. LULC refers to the composition and utilisation of the Earth’s surface, encompassing both natural and man-made features such as ecosystems, landforms, and settlements (Anandkumar et al.^3^). As defined by the Food and Agriculture Organisation (FAO), land cover refers to the physical and biological surface features of the Earth. In contrast, land use refers to the human activities and management practices associated with it. LULC change thus serves as a key indicator of human impact on ecosystems, making their monitoring essential for assessing environmental pressures, urban growth, and sustainable land management^4^. This is particularly critical in coastal regions, where these dynamic ecosystems are highly vulnerable to urbanisation, tourism, and climate change (Senthilkumar et al.^5,6^), making them sensitive to both natural and human influences^7^.

Coastal areas comprise only 10 percent of the Earth’s land but host 60 percent of the global population, leading to rapid urbanisation and economic growth^8^. This growth transforms natural landscapes into built environments, driving urban expansion that extends cities into surrounding rural areas (Mazroa et al.^9^). Land development in the coastal regions often takes the form of urban expansion and built-up areas, with concentrated economic activities such as port industries, industrial parks, and financial districts^10^. Increasing populations alter LULC patterns, affecting coastal geomorphology and shoreline dynamics, while degradation of ecosystems such as mangroves, coral reefs, and seagrass beds intensifies wave exposure and accelerates erosion^11^. Although urban growth promotes economic opportunities, it often results in the loss of agricultural land, vegetation, and natural habitats, leading to habitat fragmentation, biodiversity decline, and environmental stress^12,13^. Rising demand for land, water, and energy further accelerates resource depletion, deforestation, and pollution, undermining ecosystem stability^14^. Coastal regions are particularly vulnerable to sediment loss, shoreline shifting, seawater intrusion, landform degradation, and natural disasters like cyclones, driven by LULC changes, climate change, sea-level rise, human encroachment, and commercial expansion^15,16^. These transformations are also shaped by institutional frameworks, policies, and governance structures, reflecting the complex interplay between human activity and natural processes (Blissag et al.^17^). Understanding these dynamics is essential for assessing LULC drivers and their impacts and for devising effective strategies for sustainable land management and resource allocation.

The coastal habitats in India are drastically increasing dense population, and overall reached with 1.3 billion in 2015, causing severe LULC changes, mainly in urban coasal landscapes (Abijith & Saravanan^18,19^) India’s 7,200 km coastline is exposed to population pressures, urban encroachment, industrial discharge, and infrastructure development, while vulnerability to coastal hazards such as erosion, cyclones, and tsunamis further complicates management^20^. Kerala, located on the southwest coast, has undergone rapid urbanisation driven by high population density, rural-to-urban migration, and rising demand for housing and infrastructure, resulting in a marked shift from agricultural to non-agricultural land uses^19,21^. Several districts, including Alappuzha^22,23^, Wayanad^24^, Kozhikode^25^, and Ernakulam^26^, have reported significant land-use alterations due to human encroachment. Among them, Ernakulam and Alappuzha are of particular strategic importance, and their coastal stretches were selected for detailed analysis in this study. Regular monitoring of such changes is crucial for the sustainable management of coastal natural resources.

Geospatial technologies, such as remote sensing (RS) and geographic information systems (GIS), serve as powerful and cost-effective tools for large-scale mapping, assessment, and monitoring of natural resources using multi-resolution satellite data^27–30^. The integration of high-resolution imagery with GIS has enabled more continuous monitoring and modelling of LULC dynamics. Conventional methods, relying on field surveys, maps, and records, are time-consuming, labour-intensive, expensive, and quickly outdated in rapidly changing environments^6,31^. Cloud-based platforms such as GEE overcome these limitations by offering open-access satellite data, scalable processing, and a user-friendly web interface^32^. GEE supports cloud-based image classification using supervised machine learning algorithms such as Random Forest (RF), Support Vector Machine (SVM), and Classification and Regression Tree (CART), which offer higher accuracy and efficiency in LULC mapping compared to classical classifiers like Maximum Likelihood^33,34^. Among these, RF enhances accuracy through ensemble learning, SVM identifies optimal decision boundaries using kernel functions for high-dimensional data, and CART employs a binary tree structure to classify data based on splits of input variables^35^. Several studies have applied machine learning algorithms for LULC mapping, with many highlighting the superior performance of RF (Becker et al.^33,35,36^). Comparative studies of LULC change used RF, SVM, and CART and reported that RF and CART achieved higher accuracy than SVM (Oo et al., 2022^34^,Mangkhaseum & Hanazawa, 2021). Machine learning-based LULC classification plays a crucial role in monitoring LULC changes and analyzing future trends, as well as providing a scientific basis for sustainable land-use planning, management, and ecological restoration (Motlagh et al.^37^). Recent studies have also explored deep learning and explainable artificial intelligence (XAI) approaches for improving prediction accuracy and model interpretability in geospatial analysis^38–40^. However, conventional machine learning models such as RF continue to remain widely preferred in LULC studies due to their robustness, computational efficiency, and suitability for large-scale cloud-based platforms such as GEE.

The predictive models are generally classified into three categories, such as quantitative (Grey-Markov model—GM model), system dynamics (SD model), spatial (Cellular Automata—CA) and coupled models (CLUE-S), etc.)^4^. Coupled models, including CA–Markov, PLUS, and Land Change Modeller (LCM), integrate both quantitative and spatial aspects to simulate land-use dynamics^41^. Since the 1990s, stochastic discrete event models, including Markov chains and CA–Markov, have been among the most widely applied approaches for predicting land-use change^1,2,4,37,42,43^. The Multi-Layer Perceptron (MLP)-Markov Chain model is widely used for simulating and detecting LULC changes (Haj et al^21,44^), wherein the MLP algorithm is executed as a feedforward artificial neural network (ANN), which recognises complex nonlinear relationships, while the Markov Chain model uses transition probabilities from past observations to estimate future land-use states probabilistically^43^. LCM module in TerrSet v.20 utilises their integration to provide an effective framework for quantifying and simulating spatiotemporal LULC change patterns.

The present study focused on the coastal stretches of the Ernakulam-Alappuzha districts in Kerala, a region undergoing significant urbanisation, population pressure, and environmental change. LULC maps for 2004, 2015, and 2024 were classified using machine learning algorithms (RF, SVM, and CART), implemented on the cloud-based GEE platform. Thus, the study integrates cloud-based machine learning classification and MLP–Markov simulation within a unified geo-computational framework for long-term coastal LULC prediction, enabling improved spatio-temporal analysis and future scenario modelling in a rapidly changing coastal environment. These classifications provided baseline information for analysing historical changes and assessing LULC transformation patterns. The LCM module in TerrSet v.20 software was then used to project future LULC scenarios for 2034, 2044, and 2054. The study supports Sustainable Development Goal 11 (Sustainable Cities and Communities), particularly SDG 11.3, by providing scientific insights into urban growth and land-use transformation for sustainable coastal planning and management. This integrated approach facilitates a comprehensive understanding of past, present, and potential future LULC dynamics, providing a scientific basis for sustainable land-use planning and management in the coastal zone.

## Study area

**Fig. 1:**
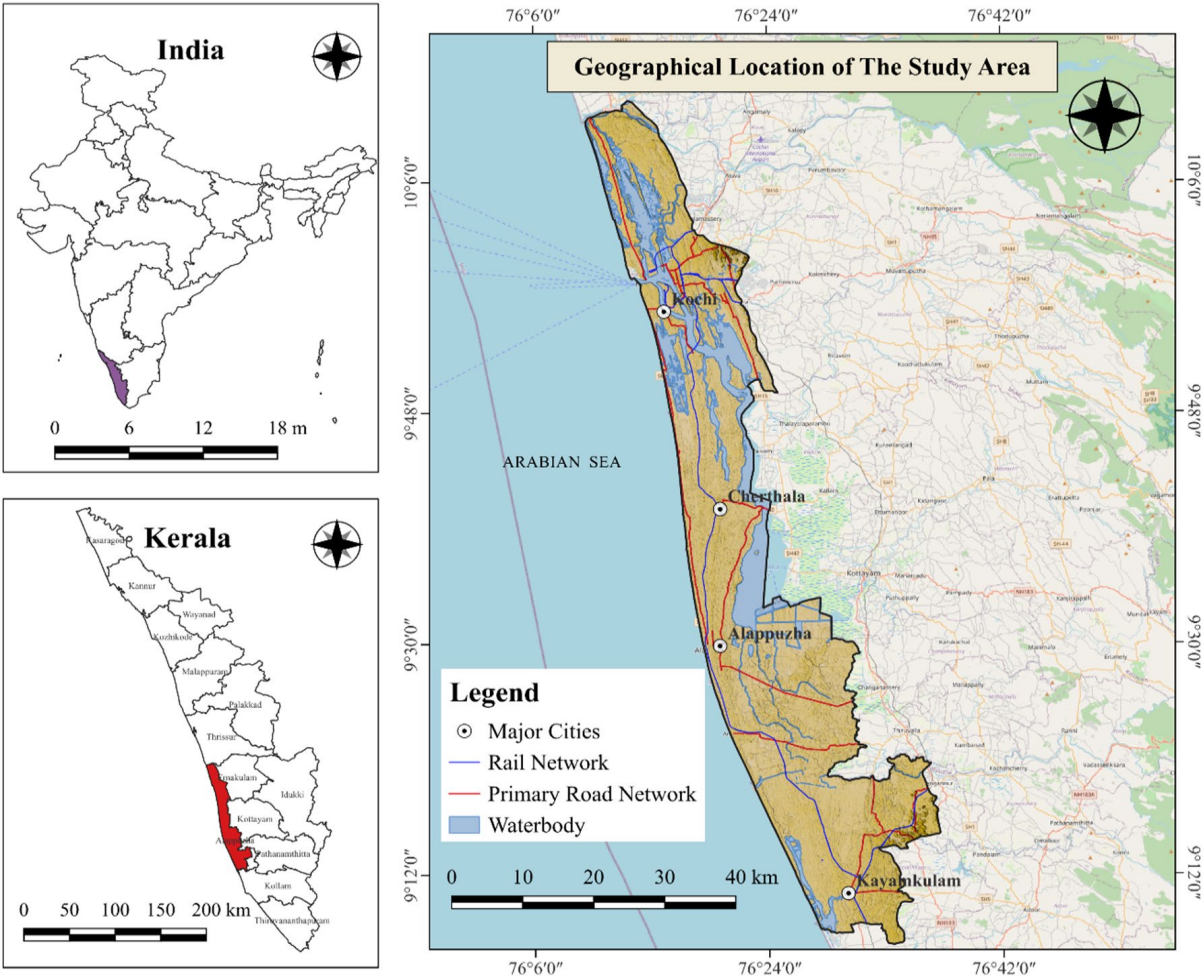
Location map of the study area (Map Prepared in QGIS 3.44—https://qgis.org/).

The study area (Fig. 1) covers the coastal stretches of Ernakulam and Alappuzha districts, representing a region of both economic and strategic importance in Kerala. Located in the southwestern part of the state along the Arabian Sea, the region supports various landuse and human activities. It lies between 9°06′ and 10°12′ N latitudes and 76°06′ and 76°42′ E longitudes. Kuttanadu, known as the “rice bowl of Kerala,” represents the region’s agricultural importance. At the same time, Kochi, the “Queen of the Arabian Sea,” serves as a key port city and district headquarters, highlighting trade, commerce, and urban growth. The combination of agricultural zones and urban-industrial centres makes this region important for studying land use, coastal management, and regional development. Ernakulam and Alappuzha districts exhibit distinct physiographic features, but this study is limited to their coastal plain regions. In Ernakulam, the coastal plain lies to the west, with low-lying areas up to 10 m in elevation, characterised by backwaters, marshes, sandy flats, and alluvial plains prone to monsoon flooding. Alappuzha is predominantly a coastal plain with elevations mostly below 6 m, including the Kuttanad deltaic region, characterised by extensive wetlands. The area features prominent coastal landforms, including beaches, shore platforms, and spits, along with major backwater systems such as Vembanadu, Karthikapally, Vayalar, and Vatta Kayals, reflecting the complex geomorphology and hydrological dynamics of the coastal plains. The study area has a tropical humid climate with hot summers and abundant rainfall, influenced by the southwest monsoon (June–September) and northeast monsoon (October–December). Alappuzha and Ernakulam receive annual rainfall of about 2965 mm and 3359 mm, respectively. March and April are the hottest months, while December and January are the coolest, with temperatures ranging from 22.6–32.7ºC in Alappuzha and 23.2–31.4ºC in Ernakulam. The drainage system supports its agriculture and ecology, with Ernakulam drained by the Periyar and Muvattupuzha Rivers, and Alappuzha by the Pamba River and its tributaries, including Achankovil and Manimala Rivers.

## Materials and methods

The study analyses LULC changes and their future simulation using a combination of satellite-derived and ancillary datasets. LULC data for 2004, 2015, and 2024 were extracted from Landsat ETM + and OLI/TIRS imagery (Table 1). The driving factors used in the LULC prediction model were selected based on their established influence on land transformation and urban growth processes reported in previous studies^38,40,45^. These variables were grouped into topographic factors (elevation, slope, and aspect) and proximity/accessibility factors (distance from roads, railways, waterbodies, and built-up areas), as these factors are widely recognized to influence settlement expansion, accessibility, and the spatial patterns of land-use change. Landsat images (path (144) and rows (53 and 54)) were selected based on two criteria: cloud cover below 5% and acquisition during the same season. The 2004 and 2024 images were captured in December, and the 2015 image in January. Although a Landsat image was available for 12 December 2014, it had about 38% cloud cover,therefore, the image from 13 January 2015, with less than 10% cloud cover and within the same agricultural season, was selected to improve image quality and classification reliability. All satellite data were acquired from the United States Geological Survey (USGS) and processed using GEE, a cloud-based platform that enables efficient analysis of decades of Earth Observation Data, including imagery from Sentinel, Landsat, MODIS, and other climate and demographic datasets (Becker et al.^33,36^). The LULC classifications for 2004, 2015, and 2024 were performed using GEE, and the resulting classified maps were subsequently used for LULC change-prediction modelling for 2034, 2044, and 2054, applied to the coastal stretch of the Ernakulam–Alappuzha districts. Accordingly, the methodology is organised into two major sections. The first section describes the LULC classification process implemented in GEE using machine learning algorithms, RF, SVM and CART, to assess and compare classification accuracy. The second section outlines the LULC change prediction modelling carried out using the Multi-Layer Perceptron–Markov Chain (MLP–MC) model within the Land Change Modeller (LCM) module of TerrSet v.20.

**Table 1:** Specifications of landsat and ancillary datasets used for the study.

Sl.No	Factor types	Potential driving factor	Description	Year
1	Landscape factors	Landuse/ Land cover	Landsat 7, 8, and 9 ETM + , OLI & TIRS images derived from Google Earth Engine & USGS Earth Explorer	2004, 2015 and 2024
		NDVI		
		NDWI		
2	Topographic factors	Elevation	AW3D Standard DEM (https://www.aw3d.jp/en/products/standard/)	Composite (2006–2011)
		Slope		
		Aspect		
3	Proximity factors	Euclidean distance from roads/rail network	Derived from the QuickOSM plugin in QGIS	2024
		Euclidean distance from built-up area	Derived from LULC 2015 and LULC 2024	2015 & 2024
		Euclidean distance from waterbody	Derived from LULC 2015 and LULC 2024	2015 & 2024

**Fig. 2:**
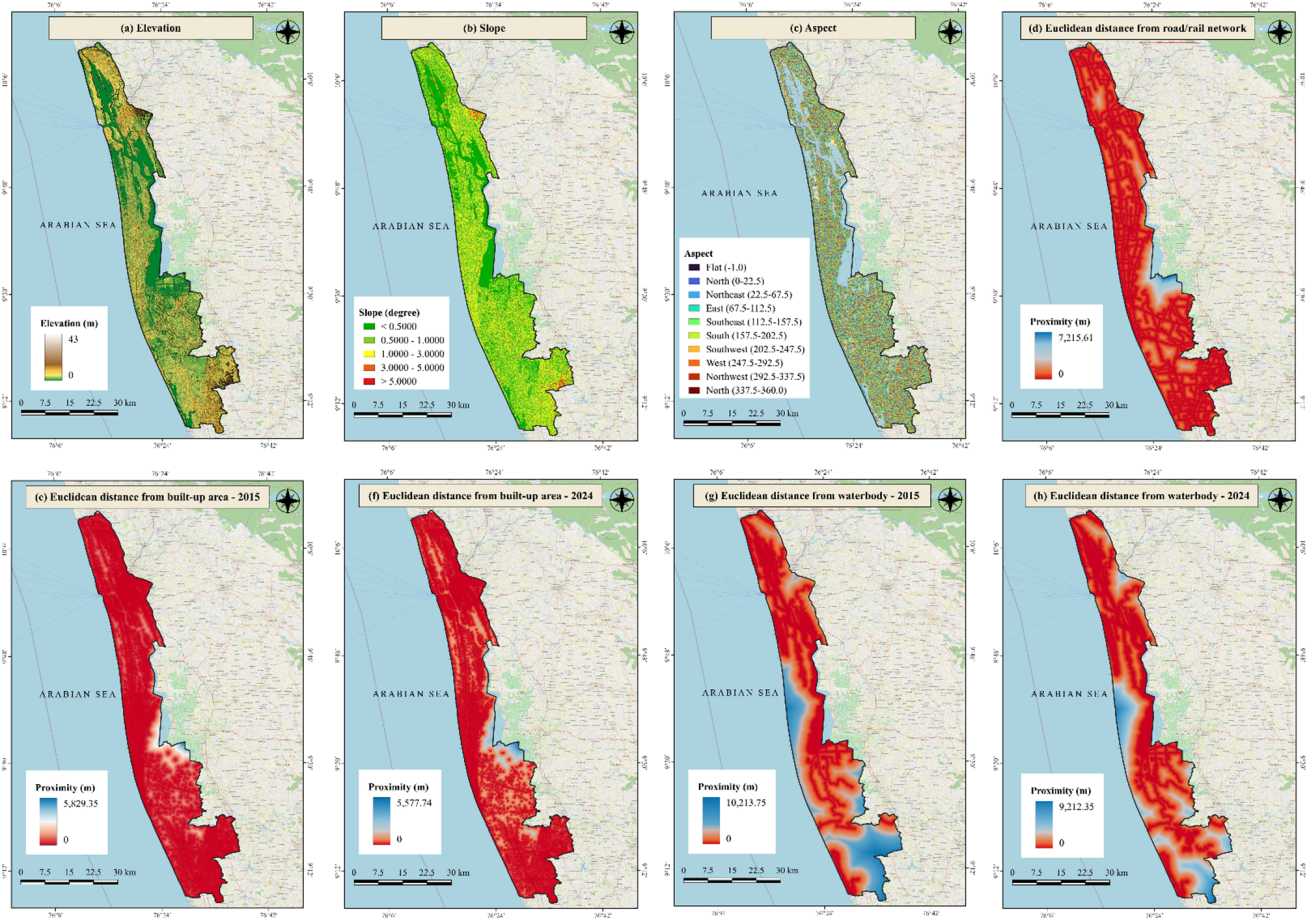
Spatial variability of driving factors considered for LULC change prediction modelling, including (**a**) elevation, (**b**) slope, (**c**) aspect, (**d**) Euclidean distance from road/rail network, (**e**–**f**) Euclidean distance from built-up areas (2015 and 2024), and (g–h) Euclidean distance from waterbodies (2015 and 2024) (Map Prepared in QGIS 3.44—https://qgis.org/).

Figure 2 illustrates the explanatory variables used in this study to simulate future LULC scenarios. Topographic data were derived from the ALOS World 3D (AW3D©JAXA) Standard DEM product at 30-m resolution, sourced from PRISM (Panchromatic Remote-Sensing Instrument for Stereo Mapping) tri-stereo imagery aboard the ALOS satellite, released by the Japan Aerospace Exploration Agency (JAXA) in 2016^46^. From this DEM, slope and aspect layers were generated. The study area is predominantly flat, with elevations ranging from 0 to 53 m, and most of the region lies between 0 and 10 m. Slope provides information on surface inclination, while aspect represents the directional orientation of that slope. Rail and road network data were obtained using the QuickOSM plugin in QGIS. LULC maps from 2015 and 2024, extracted from GEE imagery, reveal significant changes in water bodies and built-up areas. Euclidean distance tools in QGIS were then used to calculate proximity to roads/railways, water bodies, and built-up areas. These distance variables were included to account for the likelihood that new urban development tends to emerge near existing transportation infrastructure and built-up regions^41^.

**Fig. 3:**
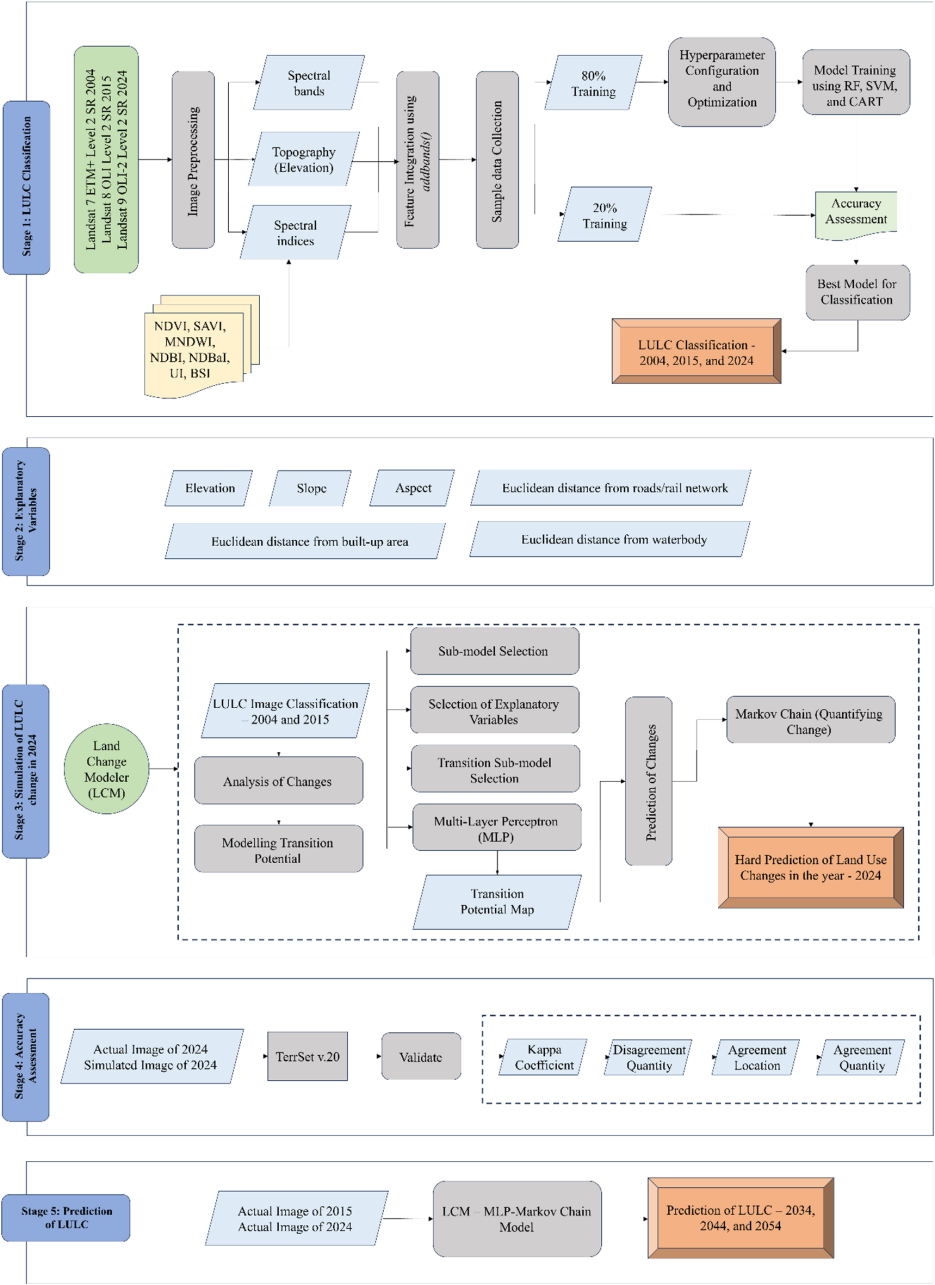
Overall methodological framework for LULC classification and prediction using machine learning and land change modeller.

Figure 3 represents the overall methodological framework adopted for LULC classification, simulation, and prediction. The workflow comprises different stages: (1) LULC classification for 2004, 2015, and 2024 using machine learning algorithms (RF, SVM, and CART); (2) simulation of LULC change using the Land Change Modeler (LCM) integrated with Multi-Layer Perceptron (MLP) and Markov Chain; (3) accuracy assessment through Kappa and agreement metrics; and (4) prediction of future LULC scenarios for 2034, 2044, and 2054.

### Image preprocessing

For 2004, a Collection 2 Level-1 Precision Terrain (L1TP) corrected Landsat 7 scene dated 08 December 2004 was acquired from the USGS Earth Explorer, and the reflective bands (Bands 1–5 and 7) of the ETM + sensor, which provides 15 m panchromatic, 30 m reflective, and 60 m thermal bands, are used for this study. Scan line errors in the six selected bands were corrected in QGIS v3.34, using inverse distance weighting interpolation followed by smoothing^47^. The gap-filled bands were then atmospherically corrected using the Dark Object Subtraction (DOS) method in the Semi-Automatic Classification Plugin (SCP). Visual inspection indicated minimal interpolation-induced linear artifacts following the gap-filling process, consistent with previous Landsat 7 SLC-off studies (Asare et al.^48^). The corrected bands were composited and clipped to the study area for the 2004 LULC classification. For 2015 and 2024, Collection 2 Level 2 surface reflectance images from the Landsat 8 and 9 Operational Land Imager (OLI) sensors were obtained from the GEE data catalogue. The data provider geometrically, radiometrically, and atmospherically corrects these images^34^. These selected 2004, 2015, and 2024 satellite images were subsequently used for LULC classification.

### Land use land cover (LULC) classification using GEE

Pre-processed satellite images with less than 5% cloud cover from 2004, 2015, and 2024 were used as the primary inputs for classification. Upon importing the data into GEE, cloud and cloud shadow removal were carried out using cloud masking techniques based on the ‘QA_PIXEL’ quality assessment band available with Landsat Surface Reflectance data^34^. Median composites for the respective years were generated to fill gaps from cloud masking. However, since the selected images had minimal cloud contamination, only a negligible amount of masking was required.

In the second phase of the methodology, in addition to the multispectral bands, several spectral indices were computed to enhance classification accuracy^34^. These included the Normalized Difference Vegetation Index (NDVI), Soil-Adjusted Vegetation Index (SAVI), Modified Normalized Difference Water Index (MNDWI), Normalized Difference Built-up Index (NDBI), Normalized Difference Bareness Index (NDBaI), Bare Soil Index (BSI), and Urban Index (UI), which highlight key land surface characteristics such as vegetation, built-up areas, bare soil, and water bodies (Table 2). Elevation was also incorporated as an auxiliary variable to improve classification performance further, giving importance to its influence on the spatial distribution of land cover types. Before sampling, the multispectral bands, spectral indices, and elevation data were integrated into a composite image.

**Table 2:** Spectral indices used for the study and their detailed description.

Spectral indices	Full name of indices	General equation	Description	Reference
NDVI	Normalized difference vegetation index	$NDVI=\frac{NIR-RED}{NIR+RED}$	Quantify vegetation growth, biomass, and changes in plant health	Tucker and Red^81^
SAVI	Soil-adjusted vegetation index	$SAVI=1.5\times \frac{NIR-RED}{NIR+RED+0.5}$	Vegetation index minimizing soil brightness influences using the soil-brightness correction factor	Huete^82^
MNDWI	Modified normalized difference water index	$MNDWI= \frac{GREEN-SWIR1}{GREEN+SWIR1}$	Enhances open water features, suppressing built-up areas and noise from other land covers	Xu^83^
NDBI	Normalized difference built-up index	$NDBI=\frac{SWIR1-NIR}{SWIR1+NIR}$	Quantify built-up or urbanized areas within a landscape	Zha et al.^84^
NDBaI	Normalised difference bareness index	$NDBaI=\frac{SWIR1-SWIR2}{SWIR1+SWIR2}$	Quantify the degree of bareness or lack of vegetation cover	Zhao and Chen^85^
BSI	Bare soil index	$BSI=\frac{\left(\left(SWIR1+RED\right)-\left(NIR+BLUE\right)\right)}{\left(\left(SWIR1+RED\right)+\left(NIR+BLUE\right)\right)}$	Enhances detection of exposed soil and uncultivated areas by combining NDVI and NDBI	Rikimaru et al.^86^
UI	Urban index	$UI=\frac{SWIR2-NIR}{SWIR2+NIR}$	Contrast between NIR and SWIR2 reflectance to enhance the detection of urban areas	(Kawamura et al., 1996)

In the next phase, training and test datasets were prepared for nine LULC classes: built-up area, sandy beach, waterbody, wetland, vegetation, cropland, barren land, fallow land, and plantation. These classes were delineated through visual interpretation of Landsat RGB false-colour composite images and historical high-resolution satellite imagery available on the Google Earth platform for defining LULC categories^49^. A sufficient number of accurate training samples was required to ensure effective classification. A total of 4496 samples for 2004, 3922 for 2015, and 5694 for 2024 were manually collected across the identified land use classes. The independence of the training and validation datasets was ensured by randomly partitioning the manually collected samples into separate training (80%) and validation (20%) subsets for each study year. The 80:20 ratio was selected as it provides sufficient data for model training while maintaining reliable validation performance^50^. Accordingly, 816, 722, and 1173 spatially independent validation points were used to assess the accuracy of the 2004, 2015, and 2024 LULC maps, respectively. The validation samples were spatially independent and were not included in the model training process. The test datasets were selected to be representative of the same spatial and temporal extent as the training data, enabling an unbiased evaluation of the model’s performance and minimizing overfitting during the accuracy assessment. The datasets were subsequently imported into GEE as a feature collection.

#### Classification algorithms

Three widely used ML classifiers, including Classification and Regression Trees (CART), Random Forest (RF), and Support Vector Machine (SVM), were implemented to derive LULC maps. When combined with geospatial techniques, CART, RF, and SVM provide tools for accurately classifying and predicting land cover classes. They effectively capture spatiotemporal changes, offering valuable insights for land management, environmental monitoring, and urban planning^51^. By integrating spatial data with machine learning, these models enhance the understanding of LULC dynamics and support informed decision-making. Breiman et al.^52^ proposed CART, a non-parametric binary classifier based on hierarchical decision trees, that iteratively split the data based on predefined thresholds until non-dividable leaf nodes were reached, which were then used as outputs for prediction^53^. The CART algorithm uses the statistical measures of Gini impurity and entropy to construct a decision tree^54^. The Gini Impurity Index selects the input feature that provides the best split at each node^55^.

1 $$G=sum\left[p\lambda \left(1-p\lambda \right)\right]$$

where $G$ represents Gini’s impurity index, $p\lambda$ is the proportion of training samples with class $\lambda$. The Gini impurity index ($G$) ranges from 0 to 1, with higher values indicating greater unevenness in the sample dataset^55^. CART offers several advantages, including easy interpretability, fast execution, and high accuracy for image classification. However, it is prone to overfitting and can produce overly complex trees that do not generalise data well. In this study, CART was implemented in GEE using the integrated *ee.Classifier.smileCart* function for LULC classification. The parameters involved in GEE include the maximum number of leaf nodes (*maxNodes*) and minimum leaf population (*minLeafPopulation*).

The RF algorithm, proposed by Breiman^56^, is one of the most widely used techniques for LULC classification with satellite imagery. As an ensemble method, RF combines multiple decision trees, assigning the final class by majority voting^57^. The responses from individual trees are evaluated, and the majority class is assigned as the final LULC class. The RF model can be defined using Eq. 2.

2 $${P}_{d}\left(i,k\right)=\frac{1}{M}{\sum}_{n=1}^{M}I\left({h}_{n}\left(x\right)=d\right)$$

where ${P}_{d}\left(i,k\right)$ is the probability of land use type $k$ at cell $i$, $d=1$ indicate a change to LULC type $k$ and $d=0$ represents other transitions, $x$ is the vector of multiple driving factors, $I$(•) is the indicator function of the decision tree set, ${h}_{n}\left(x\right)$ is the prediction type of the nth decision tree for vector $x$, and $M$ is the total count of decision trees^58^. In this study, RF was implemented in GEE using the integrated *ee.Classifier.smileRandomForest* function for LULC classification. RF classifiers typically use six input parameters in GEE: number of trees (*numberOfTrees*), number of variables per node (*variablesPerSplit*), random seed (*seed*), minimum leaf population (*minLeafPopulation*), fraction of input variables bagged per tree (*bagFraction*), maximum number of leaf nodes (*maxNodes*), and out-of-bag (OOB) mode. Factors that influence the performance of the RF model include the number of trees in the forest, the depth of individual trees, and the size of the bootstrap samples used during training^59^. The tree formation process in RF involves two steps. In the first step, each tree is built using different subsets of data and variables to enhance model performance^49^. Accuracy typically improves with more trees until it stabilises without overfitting, with optimised values selected using OOB outputs. The second step applies binary rules for node splitting, based on maximum information gain, maximum gain rate, or minimum Gini index (Eq. 1). During partitioning, node purity increases as samples within the node become more homogeneous.

SVM is a vector space-based machine learning method that determines an optimal decision boundary between classes by constructing a hyperplane^33^. SVM classifiers construct an optimal hyperplane during training that separates classes with minimal misclassification. The model can be defined using Eq. 3.

3 $$f\left(x\right)={\sum}_{i=1}^{P}{a}_{i}K(x,{x}_{i})$$

where ${a}_{i}$ is an element of the parameter vector, ${x}_{i}$ is a vector of regressors, $K$ is a function referred to as the kernel, and $P$ is the number of parameters^60^. The classifier relies on support vectors, critical data points that define the hyperplane, with parameters such as cost parameter (C), Gamma, and kernel functions controlling model performance^33^. Grid search is commonly used to optimise C and Gamma, while linear kernels are preferred for large datasets. SVM is effective for both binary and multi-class classification, where strategies like “one-vs.-one” or “one-vs.-all” are used with voting mechanisms to improve class-wise performance^49^. It performs well with limited training data due to its strong generalisation ability, though performance may degrade with high-dimensional data and insufficient samples^49,51,61^. This study implemented the SVM algorithm in GEE using the ‘ee.Classifier.libsvm’ function to classify satellite images.

#### Accuracy assessment

Accuracy assessment is an essential step for verifying the reliability of LULC maps, as it highlights potential sources of error and enhances the quality of derived information^6,47,58^. In this study, the performance of the classified images was assessed using test datasets derived from high-resolution Google Earth imagery. The classification accuracy of the LULC maps for 2004, 2015, and 2024 was evaluated using the confusion matrix approach, from which Overall Accuracy (OA), Kappa coefficient, precision, recall, and F1-score were derived. The confusion matrix compares the actual class labels with the predicted ones, where pixels located along the diagonal represent correctly classified classes, while those in the off-diagonal positions indicate misclassifications^21^. The assessment parameters employed in this study are mathematically defined and presented in Table 3.

**Table 3:** Accuracy assessment metrics and its definitions.

Accuracy Assessment Metrics	Equation	Description
Accuracy	${\mathrm{Accuracy}} = \frac{{{\mathrm{correct}}\;{\mathrm{classifications}}}}{{{\mathrm{total}}\;{\mathrm{classifications}}}}$	Proportion of all classifications that were correct, whether positive or negative
Precision	${\mathrm{Precicion}} = \frac{{{\mathrm{correctly}}\;{\mathrm{classified}}\;{\mathrm{actual}}\;{\mathrm{positives}}}}{{{\mathrm{everything}}\;{\mathrm{classified}}\;{\mathrm{as}}\;{\mathrm{positive}}}}{\text{ }}$	Proportion of all the model’s positive classifications that are actually positive
Recall	${\mathrm{Recall}}\; = \;\frac{{{\mathrm{correctly}}\;{\mathrm{classified}}\;{\mathrm{actual}}\;{\mathrm{positives}}}}{{{\mathrm{all}}\;{\mathrm{actual}}\;{\mathrm{positives}}}}$	Proportion of all actual positives that were classified correctly as positives
F1-score	$F1 - {\mathrm{Score}} = 2 \times \frac{{{\mathrm{Precision}}\; \times \;{\mathrm{Recall}}}}{{{\mathrm{Precision}}\; + \;{\mathrm{Recall}}}}$	Harmonic mean between precision and recall
Kappa Coefficient	${\mathrm{Kappa}} = \frac{{\left( {{\mathrm{TS}} \times {\mathrm{TCS}}} \right) - \sum {\left( {{\mathrm{Column}}\;{\mathrm{total}}\; \times \;{\mathrm{Row}}\;{\mathrm{total}}} \right)} }}{{{\mathrm{TS}}^{2} - \sum {\left( {{\mathrm{Column}}\;{\mathrm{total}} \times {\mathrm{Row}}\;{\mathrm{total}}} \right)} }}$	Degree of agreement between the predicted labels and the ground truth

#### Classification strategy

To determine the most suitable ML algorithm for LULC classification in the study area, tests were conducted using 2015 as a reference. From 3,922 samples generated through visual interpretation of high-resolution GEE imagery, 3,200 were used for training and 722 for testing. The classification methods (CART, RF, and SVM) were implemented directly in GEE, with a grid search hyperparameter optimisation approach through iterative testing of predefined value ranges informed by existing literature^49^. The best-performing hyperparameter combination was selected for each algorithm based on accuracy, as summarised in Table 4.

**Table 4:** Hyperparameter Settings for the 2015 LULC Classification.

Sl. No	ML Algorithm	Hyperparameters	2015 Values
1	CART	maxNodes	null (*unlimited nodes*)
		minLeafPopulation	5
2	RF	numberOfTrees	300
		variablesPerSplit	null (*square root of number of variables*)
		minLeafPopulation	1
		bagFraction	0.5
		maxNodes	null (*unlimited nodes*)
		seed	0
3	SVM	cost	100
		kernelType	Linear

**Fig. 4:**
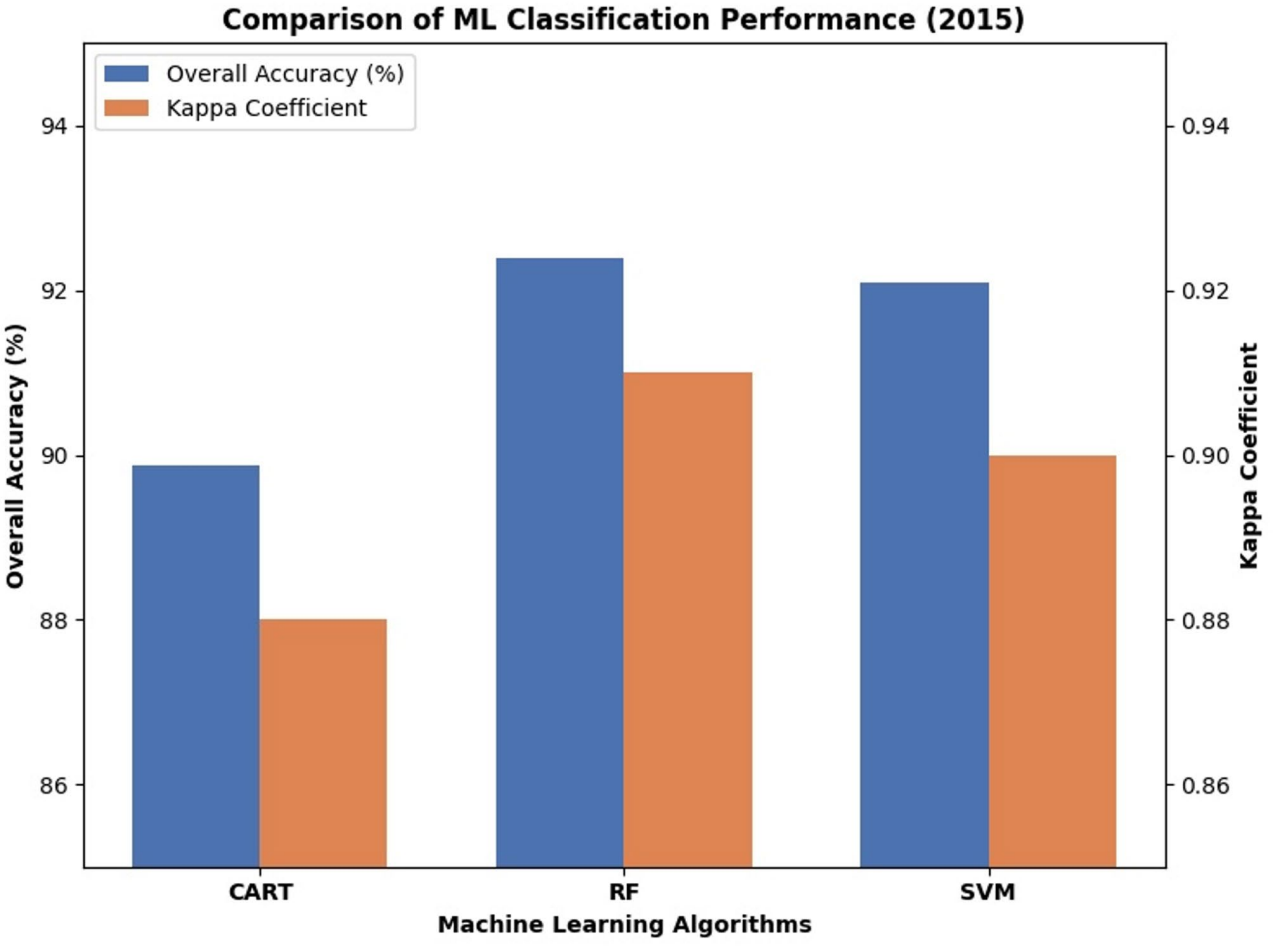
Comparison of ML classification performance (2015).

The RF classifier achieved the highest performance, with an overall accuracy of 92.38% and a Kappa coefficient of 0.91 (Table 5 and Fig. 4). Figure 4 visually illustrates the comparative overall accuracy and Kappa coefficients of the CART, RF, and SVM algorithms, highlighting the superior classification efficiency of RF. The high overall accuracy indicates that RF correctly classified most land cover types, while the Kappa value reflects a high level of agreement with reference data. This demonstrates that RF is particularly well-suited for LULC classification in regions with complex geomorphology and heterogeneous landscapes, owing to its ability to manage high-dimensional data and capture intricate spectral relationships^12,33^. SVM also performed strongly, achieving 92.10% accuracy and a Kappa of 0.90. Although slightly lower than RF, these results demonstrate substantial agreement, highlighting SVM’s effectiveness for complex classification tasks. CART, with 89.88% accuracy and 0.88 Kappa, produced reliable but comparatively weaker results, suggesting limitations in handling the complexity of this LULC task.

**Table 5:** Results of testing different classification methods.

Classification method	2015	
	Overall accuracy (%)	Kappa coefficient
CART	89.88	0.88
RF	92.38	0.91
SVM	92.10	0.90

Based on these findings, RF was selected as the primary classification algorithm for this research. For 2004 and 2024, RF was implemented using separately optimised hyperparameters obtained through independent grid search analyses. The optimisation results indicated variation in the optimal *numberOfTrees* parameter across datasets, with *numberOfTrees* = 300 selected for 2004 and *numberOfTrees* = 100 applied for 2024. For both years, the remaining parameters were identical to those optimised for 2015. In the post-processing stage, the LULC classification results still exhibited small, fragmented patches due to land surface heterogeneity^12^. To address this, the sieve tool in QGIS was applied to smooth the images by removing raster polygons smaller than a specified threshold (3 pixels in this study) and replacing them with the value of the largest neighbouring polygon. The final classification outputs were validated using multiple accuracy metrics to assess model performance.

### MLP- Markov chain-based land change modeller

The Land Change Modeller (LCM) within the TerrSet v.20 Geospatial Monitoring and Modelling System (TGMMS) was employed to predict future LULC scenarios for the years 2034, 2044, and 2054 using classified historical satellite imagery. TerrSet v.20, developed by Clark Labs at Clark University, is an integrated geospatial suite for earth system monitoring and sustainable development, with its Land Change Modeller (LCM) enabling analysis of land transformation and biodiversity conservation (Eastman, 1987). It includes the “Change Analysis” module to detect variations across time periods, the “Transition Potentials” module to assess areas likely to change, and the “Change Prediction” module to forecast future LULC classes^1^.

#### LULC change analysis

The first step in the prediction process is Change Analysis, which evaluates transitions between two land cover maps from time 1 to time 2, representing shifts from one land cover type to another, with the number of possible transitions determined by the total number of LULC classes^42^. In this study, Change Analysis was performed by comparing classified images for 2004–2015, 2015–2024, and 2004–2024 to identify the dominant land cover transitions. The cross-tabulation analysis was performed to quantify the changes during these periods. The Change Analysis tab enabled rapid assessment of gains, losses, net change, persistence, and specific transitions through maps and graphs, providing category-wise summaries of LULC change. To enhance accuracy and focus on the most significant transitions, dominant transitions were limited to nine, and the Ignore Transitions option was used to exclude minor or insignificant changes. Accordingly, land cover changes smaller than 20 sq. km for 2004–2015, 27 sq. km for 2015–2024, and 24 sq. km for 2004–2024 were ignored, representing less than 2% of the total study area. This refinement ensured that only the most relevant transitions were included in the Transition Potential analysis. Probability matrices were generated for 2004–2015, 2015–2024, 2024–2034, 2034–2044, and 2044–2054 using LCM, and the change percentage and rate of change for each LULC category were calculated using Eqs. (4) and (5).^2^

4 $$\mathrm{Percentage}\,\mathrm{of}\,\mathrm{change} = \frac{{A}_{y}-{A}_{x}}{{A}_{x}}\times 100$$

5 $${\mathrm{Rate}}\;{\mathrm{of}}\;{\mathrm{change}}\left( {{\mathrm{sq}}.{\mathrm{km}}/{\mathrm{year}}} \right) = \frac{{A_{y} - A_{x} }}{T}$$

where ${A}_{x}$ is the area of a LULC class (sq.km) in the earlier land cover image, ${A}_{y}$ is the area of the same class in later image, $T$ is the time interval between the two images in years.

#### Transition potential modelling

The second step in the prediction process, Transition Potential Modelling, aims to identify areas most likely to undergo land cover change by analysing the spatial relationships between land cover transitions and their driving variables (ancillary datasets)^43^. Each sub-model estimates the probability of conversion from one land cover class to another during the study period. Based on the major transitions observed in the study area, multiple sub-models were generated, with optimal thresholds applied to exclude minor transitions representing less than 2% of the total area (Motlagh et al.,^37^). The analysis focused on dominant transitions that consistently influenced land cover dynamics across the study periods. For 2004–2015, major conversions included transformations from plantation to built-up and vegetation, as well as wetland-related changes to waterbody, vegetation, cropland, and plantation. During 2015–2024, similar patterns persisted, with notable transitions involving plantation and wetland conversions to built-up, vegetation, and cropland classes. Over the extended period 2004–2024, these trends remained dominant, highlighting plantation, wetland, and vegetation interactions as the primary drivers of land cover change in the study area. Each transition produces a potential map representing its time-specific likelihood of change.

For each transition sub-model, a corresponding set of explanatory (driver) variables was defined and processed within the Transition Sub-Model Structure panel. These variables were categorised as static or dynamic based on their temporal characteristics (Eastman, 1987). In this study, the static variables representing site conditions that remain constant over time included elevation, slope, aspect, proximity to waterbodies, and proximity to road and railway networks. The dynamic variable, proximity to built-up areas, captured the changing influence of urban expansion on surrounding land uses. During model setup, this variable was configured using a land–use–based reference layer, allowing the model to calculate the distance from built-up areas for each period. This integration ensured that temporal variations in urban growth were effectively incorporated into the transition potential modelling, enhancing the accuracy of land cover change prediction (Eastman, 1987). Before analysing the LULC transitions across different time periods, the influence of various driving variables on LULC changes was assessed using Cramer’s V statistic. Cramer’s V is a statistic that transforms the Chi-square value (for a contingency table larger than two rows × two columns) into a range of 0–1, where a value of 1 indicates complete agreement between the two nominal variables^62^. This measure evaluates the strength of association between driver variables and LULC changes based on Chi-square values^63^. Values between 0.15 and 0.4 indicate a moderate influence of the variables on land-use changes and are considered acceptable. Values above 0.4 suggest a strong association between change patterns and driver variables, while those below 0.1 indicate negligible influence and are excluded from the model. In this analysis, the transitions between LULC maps (2004 & 2015 and 2015 & 2024) were represented as Boolean maps, and the driver variables (scaled between 0 and 255) were used as inputs.

The Run Transition Sub-Model panel executes the actual modelling of land cover transitions. Among the six available modelling approaches, Multi-Layer Perceptron (MLP), Decision Forest, Logistic Regression, Weighted Normalized Likelihood (WNL), Support Vector Machine (SVM), and SimWeight, the MLP neural network were used in this study. As a practical form of artificial neural network (ANN), MLP effectively captures nonlinear patterns and provides accurate mappings between dependent and independent variables (Haj et al.^37,44^). It is suitable for simulating multiple transition types and projecting land areas likely to change over time^2^. The MLP model represents complex landscape dynamics through a network of interconnected neurons organized into input, hidden, and output layers, where each neuron processes weighted inputs and transfers the results through an activation function^1^. The backpropagation algorithm is employed to train these neurons by iteratively adjusting the internal weights to minimize prediction errors, enabling the hidden layers to capture nonlinear relationships among multiple explanatory variables influencing land cover transitions^4^. This operation can be expressed mathematically as Eq. 6^64^.

6 $$y=\varphi \left({\sum}_{i=1}^{n}{w}_{i}{x}_{i}+b\right)=\varphi \left({w}^{T}x+b\right)$$

where $y$ is the output of the neuron, $w$ is the vector of weights, $x$ is the vector of inputs, $b$ is the bias and $\varphi$ is the activation function. The activation function can be either logistic sigmoid $(1/\left(1+{e}^{-x}\right))$ or hyperbolic tangent *tanh(x)*^64^. MLP requires samples of pixels that underwent each transition, as well as persistence samples—pixels eligible for change but unchanged—ideally with similar sample sizes. By default, the sample size equals the smallest transition category, capped at 10,000 pixels if exceeded. The model operates with pre-configured parameters, such as the number of hidden layer nodes and learning rate, both automatically adjusted during training to enhance performance. Ideally, the training and testing RMS (Root Mean Square) error curves show a gradual decline and approach asymptotic convergence, indicating stable learning behaviour. After extensive training iterations (10,000), the model achieved high predictive accuracy. Upon completion, an output report summarizes model performance, highlights the relative influence of explanatory variables, and generates transition potential maps illustrating the spatial likelihood of future land cover change.

#### Change prediction

Finally, the Change Prediction module integrates transition potential maps derived from the classified LULC maps of 2004, 2015, and 2024 to simulate future land cover. The extent of LULC change was modelled using the Markov Chain algorithm, a stochastic process that estimates transition quantities based on the system’s current state and transition probabilities^4^. Within TerrSet v.20 LCM, the MARKOV module uses earlier and later land cover maps to project expected transitions, accounting for equilibrium effects among competing land cover types. The process generates a transition probability matrix, transition area matrix, and conditional probability images, representing the likelihood and spatial distribution of future changes. In this study, LULC maps from 2004 and 2015 were used to predict 2024, validated against the classified 2024 map. The validated model was then used to predict future scenarios for 2034, 2044, and 2054, providing a spatially explicit understanding of long-term land cover dynamics. However, the Markov approach has a notable limitation in spatial allocation, as it models transition quantities effectively but lacks spatially explicit predictive capability^2,4,65^, and the equation (Eq. 7) is used to predict the land use change trends in future scenarios.

7 $$S\left(t+1\right)={P}_{ij}\times S\left(t\right)$$

where, $S\left(t\right)$ is the state of the system at time $(t)$, $S\left(t+1\right)$ is the state at time $\left(t+1\right)$, ${P}_{ij}$ , which denotes the transition probability matrix, which is calculated using Eq. 8^2,4,65^.

8 $${P}_{ij}=\left(\begin{array}{ccc}{P}_{11}& {P}_{12}\cdots & {P}_{1n}\\ {P}_{21}& {P}_{22}\cdots & {P}_{2n}\\ {P}_{n1}& {P}_{n2}\cdots & {P}_{nn}\end{array}\right) \mathrm{s}.\mathrm{t} \left(0\le {P}_{ij}<1 and {\sum}_{j=1}^{n}{P}_{ij}=1, \left(i,j=\mathrm{1,2},\cdots ,n\right)\right)$$

where ${P}_{ij}$ refers to the transition probability from land use $i$ to $j,$ and $n$ is the number of land uses.

In the final stage of land use change prediction, outputs from the modelling phase are used to calculate transition probabilities between land cover classes via the Markov Chain, producing hard and soft predictions. A hard prediction generates a definitive land cover map, using TerrSet’s Multi-Objective Land Allocation (MOLA) module, which resolves conflicts among land allocation objectives, prioritizes land classes, and assigns pixels with the highest probability of conversion to target classes (Motlagh et al.^37^). In contrast, a soft prediction maps vulnerability to change, aggregating transition potentials to show areas with suitable conditions for transition. By default, all modelled transitions are considered, though specific transitions can be selected to focus on specific land cover dynamics. Two aggregation options are available: maximum, assigning each pixel the highest transition probability, and logical OR, reflecting cumulative vulnerability when multiple transitions target the same location. Both hard and soft predictions were applied to generate projected LULC maps for the years 2024, 2034, 2044, and 2054, providing a comprehensive understanding of the future land cover dynamics.

#### Model validation

Validation assesses the predicted LULC map against a reference^2^. Landsat images from 2004 and 2015 were used to simulate the 2024 LULC, which was then compared with the actual 2024 map. Two approaches, visual and statistical, were applied in this study. For the visual approach, LCM was calibrated using the 2004 and 2015 maps, and validation involved a three-way cross-tabulation of the 2015 map, predicted 2024 map, and observed 2024 map. The resulting map highlights correctly predicted areas (“hits”), areas predicted to change but did not (“false alarms”), and areas that changed but were not predicted (“misses”)^2^. Once the model’s predictive capacity was verified for 2024, the simulation process was extended to project LULC maps for 2034, 2044, and 2054 using the 2015 and 2024 classified maps.

The statistical approach evaluates agreement between predicted and reference maps using the Kappa index to assess overall accuracy between the two reference maps. While the original Kappa coefficient provides overall agreement, it does not distinguish between quantity and location errors. This limitation is addressed using cause-dependent K-indices: Kno (no information), Klocation (spatial accuracy), Kstandard (ratio of misallocations by chance), and KlocationStrata (accuracy within strata)^1^. While the standard Kappa index provides a general measure of model validity, it is typically considered valid if the Kappa value exceeds 70%^66^. Together, these indices provide a comprehensive evaluation of both the quantity and spatial allocation of LULC changes^2^. Additional statistics, such as AgreementQuantity, AgreementChance, AgreementGridCell, DisagreementGridCell, and DisagreementQuantity, quantify the strength of agreement and highlight errors in proportions or spatial allocation^1^. Disagreement by quantity reflects differences in overall category proportions, while allocation disagreement captures misplacement of land cover classes, offering a detailed understanding of the model’s predictive performance^2^.

## Results and discussion

### RF-based LULC classification

LULC classes for 2004, 2015, and 2024 were extracted using the Random Forest (RF) algorithm in GEE, which integrated data acquisition, pre-processing, classification, and accuracy assessment. The analysis revealed nine primary LULC categories in the study area, namely built-up area, sandy beach, waterbody, wetland, vegetation, cropland, barren land, fallow land, and plantation, covering a total area of 1,701.8 km^2^ (170,187.727 ha).

**Fig. 5:**
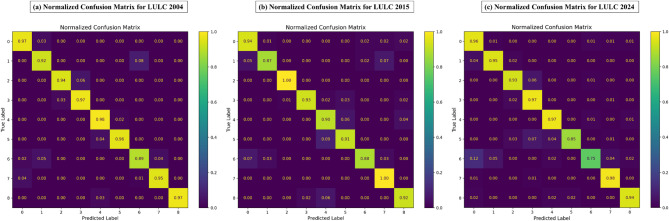
Normalized confusion matrices for the years (**a**) 2004, (**b**) 2015, and (**c**) 2024.

The accuracy of the LULC maps was evaluated using independent validation datasets, comprising 816, 722, and 1173 points for 2004, 2015, and 2024, respectively. The classification accuracy was assessed through Overall Accuracy (OA), Kappa coefficient, precision, recall, and F1-score (Table 6). Figure 5 presents the confusion matrices for 2004, 2015, and 2024, illustrating the agreement between reference data and classified results. The OA values were 95.46% for 2004, 92.38% for 2015, and 93.94% for 2024, while the corresponding Kappa coefficients were 0.95, 0.91, and 0.92. The highest classification performance was achieved in 2004, while the lowest accuracy was recorded in 2015. The consistently high OA and Kappa values across all three years indicate that the RF-based classifications are highly reliable and show strong agreement with reference data.

**Table 6:** Accuracy assessment metrics of LULC classification for 2004, 2015, and 2024.

Year	Metrics	LULC classes									Overall accuracy (%)	Kappa
		Built-up area	Sandy beach	Water body	Wetland	Vegetation	Crop land	Barren land	Fallow land	Plantation		
2004	Precision	93.62	87.27	95.71	96.55	94.74	98.53	93.51	96.94	100	95.46	0.95
	Recall	96.70	92.31	94.36	97.39	98.44	95.71	88.89	95.00	97.37		
	F1-score	95.13	89.72	95.03	96.97	96.55	97.10	91.14	95.96	98.67		
2015	Precision	93.75	94.54	98.68	99.12	91.52	82.25	95.52	82.97	81.82	92.38	0.91
	Recall	93.75	86.67	100	92.62	90.00	91.07	87.67	100	91.84		
	F1-score	93.75	90.43	99.34	95.76	90.75	86.44	91.43	90.70	86.54		
2024	Precision	96.56	88.13	93.07	90.57	95.45	99.00	84.00	93.96	89.28	93.94	0.93
	Recall	96.05	94.54	93.07	97.29	97.35	85.34	72.41	98.19	94.34		
	F1-score	96.31	91.23	93.07	93.81	96.39	91.67	77.78	96.03	91.74

Further insights into classifier performance are provided by precision, recall, and F1-scores (Table 6). In 2004, the classification results were highly balanced, with most classes achieving both high precision and recall values above 90%, resulting in good F1-scores across categories. Built-up (precision 93.62%, recall 96.70%, F1-score 95.13%), waterbody (95.71%, 94.36%, 95.03%), vegetation (94.74%, 98.44%, 96.55%), wetland (96.55%, 97.39%, 96.97%), and fallow land (96.94%, 95.00%, 95.96%) with minimal misclassification, while barren land (93.51%, 88.89%, 91.14%) recorded slightly lower recall suggesting some confusion with neighbouring classes. Plantation (100.00%, 97.37%, 98.67%) and cropland (98.53%, 95.71%, 97.10%) achieved near-perfect classification, whereas sandy beach performed comparatively lower (87.27%, 92.31%, 89.72%) due to spectral overlap. By 2015, precision remained high for several classes, including wetland (99.12%), waterbody (98.68%), barren land (95.52%), sandy beach (94.54%), built-up area (93.75%), and vegetation (91.52%). Waterbody achieved perfect recall (100%) and the highest F1-score (99.34%), confirming accurate classification. However, sandy beach (recall 86.67%, F1-score 90.43%) and barren land (recall 87.67%, F1-score 91.43%) experienced reduced recall, suggesting some misclassifications. Cropland (precision 82.25%, recall 91.07%, F1-score 86.44%) and plantation (81.82%, 91.84%, 86.54%) showed lower performance, indicating classification challenges for specific land covers during this year. In 2024, classification stability improved again, with vegetation (precision 95.45%, recall 97.35%, F1-score 96.39%), built-up area (96.56%, 96.05%, 96.31%), and fallow land (93.96%, 98.19%, 96.03%) achieving consistently strong results. Waterbody (93.07%, 93.07%, 93.07%), wetland (90.57%, 97.29%, 93.81%), and plantation (89.28%, 94.34%, 91.74%) maintained balanced accuracies, while cropland (99.00%, 85.34%, 91.67%) showed excellent precision but reduced recall. Sandy beach (88.13%, 94.54%, 91.23%) achieved moderate classification, whereas barren land remained the most challenging class with lower precision and recall (84.00%, 72.41%, 77.78%). These class-level performances are reflected in the overall accuracy and kappa values, which were highest in 2004 (95.46% and 0.95), declined in 2015 (92.38% and 0.91) due to class-specific misclassifications, and improved again in 2024 (93.94% and 0.93) with more stable results across categories.

**Fig. 6:**
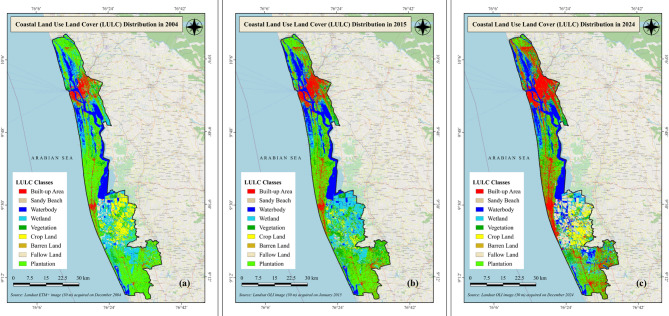
Coastal land use/land cover distribution of (**a**) 2004, (**b**) 2015, and (**c**) 2024 (Map Prepared in QGIS 3.44—https://qgis.org/).

**Fig. 7:**
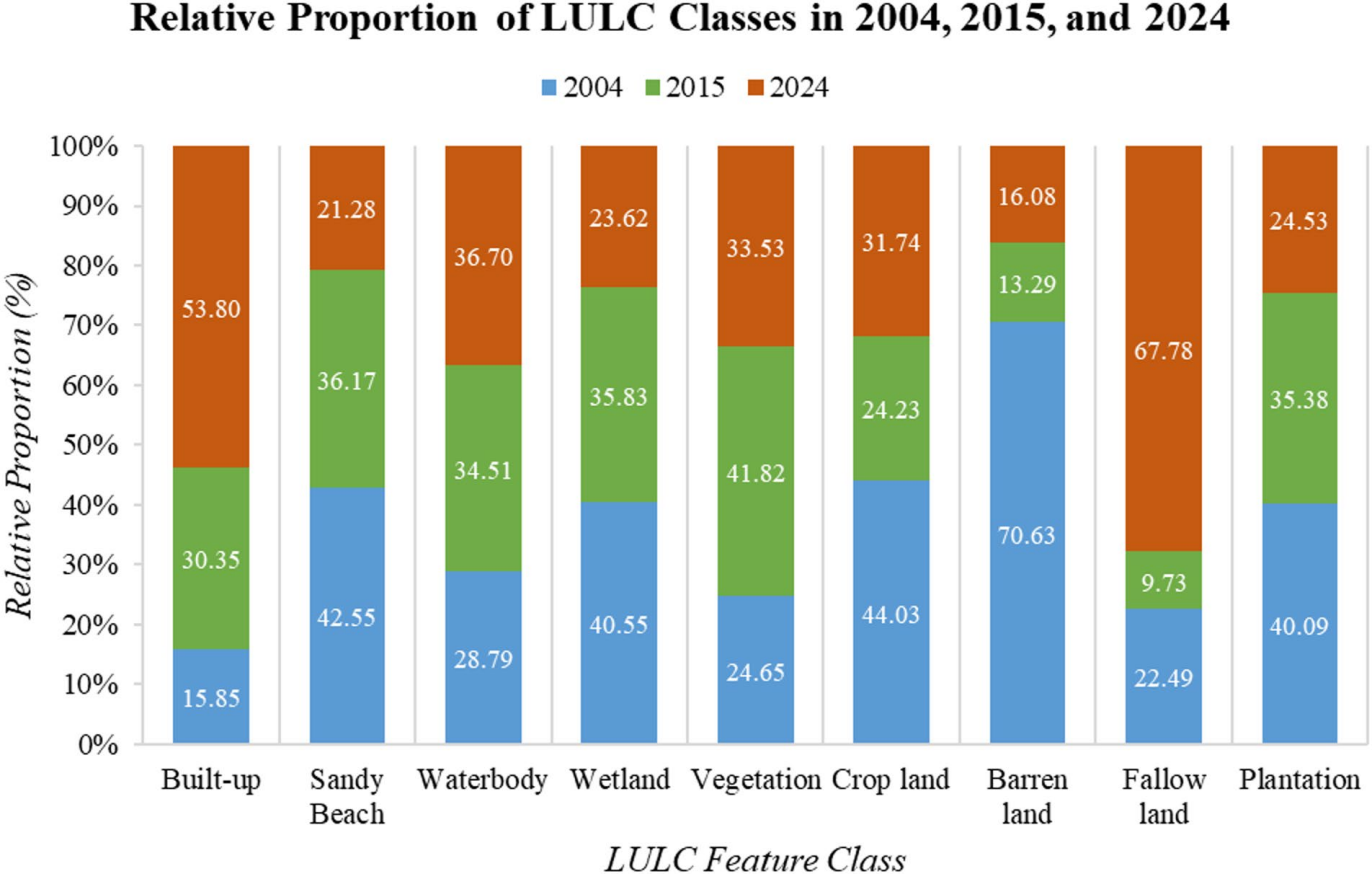
Relative percentage distribution of Land Use/Land Cover (LULC) classes for the years 2004, 2015, and 2024. (The 100% stacked column chart highlights the proportional contribution of each year within individual LULC categories.)

Figure 6 displays the LULC distribution across the nine classes for 2004, 2015, and 2024, while Table 7 presents the corresponding area statistics and annual rates of change. The analysis highlights significant land use transitions between 2004 and 2024, primarily driven by rapid urban expansion. Built-up area increased steadily from 109.93 km^2^ in 2004 to 210.58 km^2^ in 2015, and to 373.49 km^2^ in 2024. By 2024, built-up areas covered 373.49 km^2^ (22% of the study area), rising sharply from just 6.47% in 2004. In contrast, plantation exhibited the highest depletion, declining from 648.94 km^2^ in 2004 to 572.38 km^2^ in 2015, and further to 397.03 km^2^ in 2024. While the plantation occupied 38.17% of the total surface area in 2004, it shrank to 23.36% in 2024. Sandy beach also showed a consistent reduction, from 3.46 km^2^ in 2004 (0.20% of total area) to 2.93 km^2^ in 2015 and 1.67 km^2^ in 2024 (0.10%). Waterbody increased moderately, from 221.42 km^2^ in 2004 (13.02% of total area) to 265.47 km^2^ in 2015 and 282.11 km^2^ in 2024 (16.60%). Wetland area, however, declined steadily from 384.68 km^2^ in 2004 (22.63%) to 340.03 km^2^ in 2015 and further to 224.16 km^2^ in 2024 (13.19%). Vegetation increased during the first decade, expanding from 126.12 km^2^ in 2004 (7.42%) to 214.09 km^2^ in 2015 (12.59%), but later decreased to 171.66 km^2^ (10.10%). Barren land declined sharply from 34.28 km^2^ in 2004 (2.02%) to 6.53 km^2^ in 2015, before slightly increasing to 7.79 km^2^ in 2024 (0.46%). The trends in cropland and fallow land are less directly comparable due to differences in image acquisition dates and the cyclical agricultural practices followed in the Kuttanadu and coastal Alappuzha regions. The 2004 LULC map (December) recorded 119.81 km^2^ of cropland and 51.45 km^2^ of fallow land, as most cultivable areas were under active cultivation, while some fields remained inundated and were classified as wetland. The 2015 LULC map, derived from a January image (selected due to excessive cloud cover in December 2014), showed reduced cropland (65.90 km^2^) and fallow land (22.19 km^2^), as many agricultural fields were temporarily inundated during land preparation and irrigation stages and were therefore classified as wetland. In the study area, paddy cultivation commonly involves cycles of leaving fields fallow after harvest, controlled flooding to remove weeds and prepare the land, subsequent drainage before sowing, and irrigation during crop growth. Consequently, even images acquired within a short temporal interval may capture substantially different surface conditions. In 2024, using a December image, cropland covered 86.34 km^2^, while fallow land expanded to 155.13 km^2^, reflecting the practice of maintaining fields bare before the commencement of cultivation. The proportional changes illustrated in Fig. 7 strengthen these findings by highlighting the dominance of built-up growth and plantation decline, along with the marked reductions in sandy beach and wetland, moderate shifts in vegetation and fallow land, minor variations in cropland, and fluctuations in barren land. Together, the area statistics and proportional distribution provide an extensive understanding of the spatiotemporal dynamics of LULC in the study area.

**Table 7:** Area statistics and annual rate of change of different LULC features during 2004–2024.

Land use and land cover feature	2004		2015		2024	
	Area (sq. km.)	Percentage (%)	Area (sq. km.)	Percentage (%)	Area (sq. km.)	Percentage (%)
Built-up	109.93	6.47	210.58	12.39	373.49	21.98
Sandy Beach	3.46	0.20	2.93	0.17	1.67	0.10
Waterbody	221.42	13.02	265.47	15.61	282.11	16.60
Wetland	384.68	22.63	340.03	20.00	224.16	13.19
Vegetation	126.12	7.42	214.09	12.59	171.66	10.10
Crop land	119.81	7.05	65.90	3.88	86.34	5.08
Barren land	34.28	2.02	6.53	0.38	7.79	0.46
Fallow land	51.45	3.03	22.19	1.31	155.13	9.13
Plantation	648.94	38.17	572.38	33.67	397.03	23.36

### Spatiotemporal change analysis (2004–2024)

**Fig. 8:**
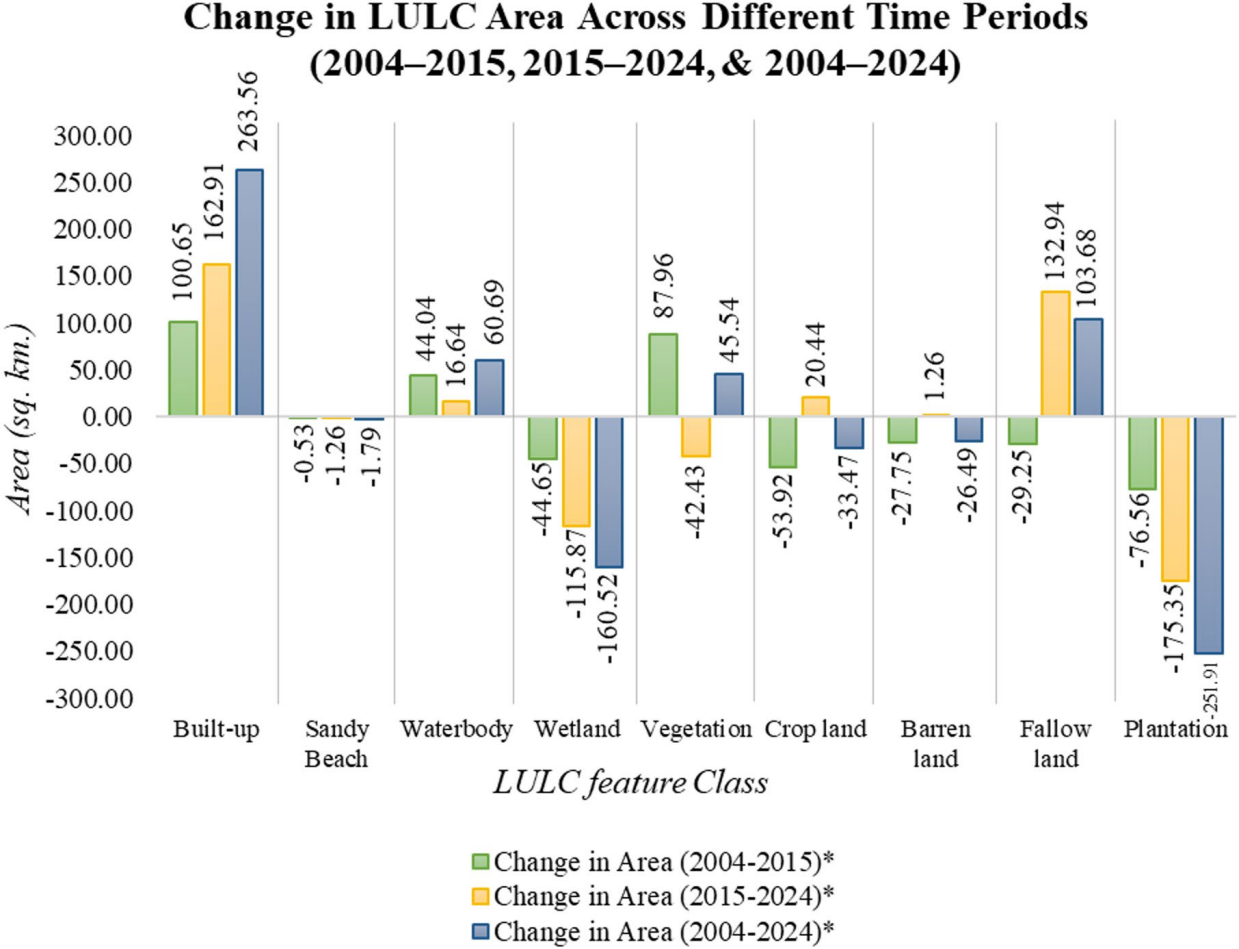
Temporal LULC area changes (2004–2015, 2015–2024, and 2004–2024) showing class-wise gains and losses.

**Fig. 9:**
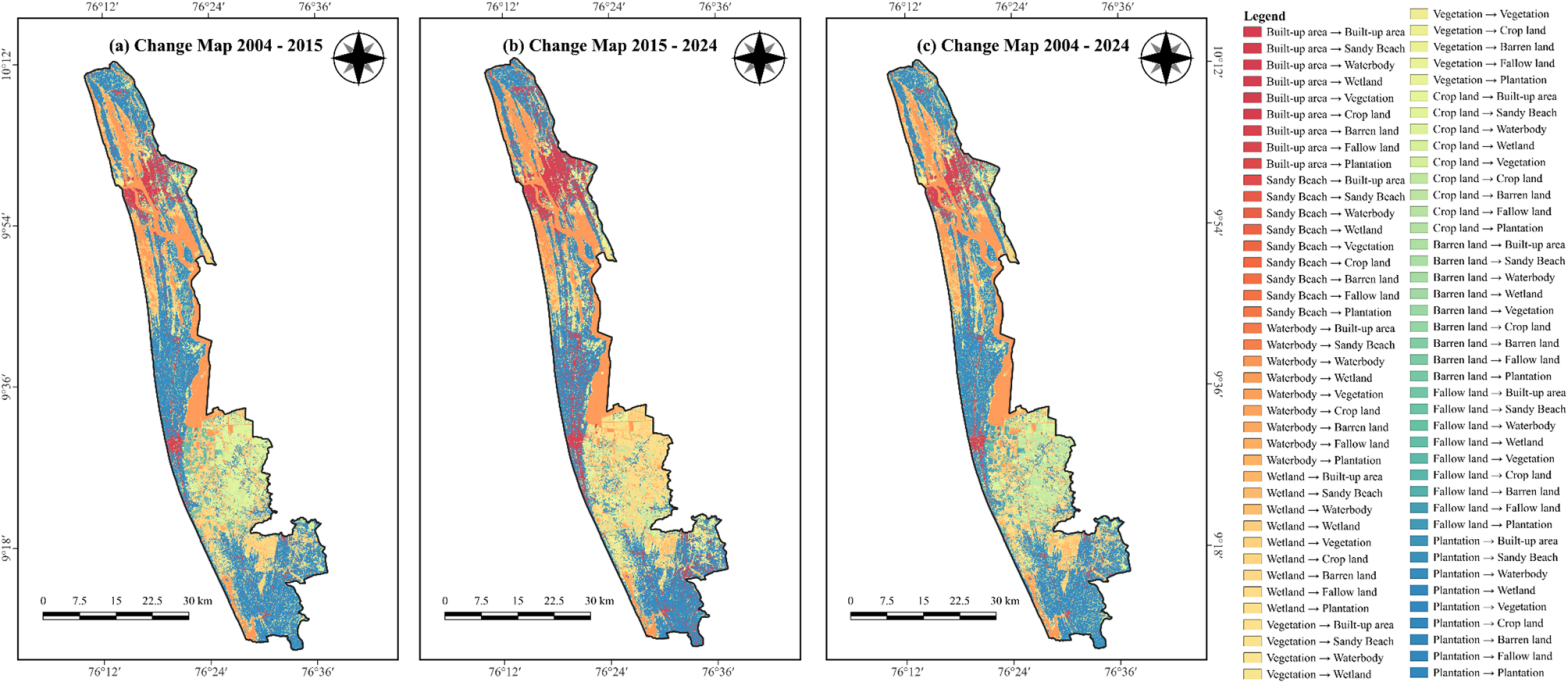
LULC change maps of the study area showing transitions between major land cover classes for (**a**) 2004–2015, (**b**) 2015–2024, and (**c**) 2004–2024 (Map Prepared in QGIS 3.44—https://qgis.org/).

To capture the fluctuating alterations in LULC types, annual variations were calculated for the periods 2004–2015, 2015–2024, and the overall 2004–2024, as shown in Fig. 8. Table 8 summarizes the area, percentage, and rate of change across all LULC categories, highlighting distinct spatial and temporal transitions during the study period. Figure 9 presents LULC change maps of the study area, illustrating transitions between major land cover classes for the periods 2004–2015, 2015–2024, and the overall 2004–2024, representing the evolution of the landscape over time.

**Table 8:** Spatiotemporal changes in LULC area, percentage, and rate of change during 2004–2015, 2015–2024, and 2004–2024.

Land use and land cover feature	Change in area (2004–2015) *			Change in area (2015–2024) *			Change in area (2004–2024) *		
	Area (sq. km.)	Percentage of change (%)	Rate of change (sq.km/year)	Area (sq. km.)	Percentage of change (%)	Rate of change (sq.km/year)	Area (sq. km.)	Percentage of change (%)	Rate of change (sq.km/year)
Built-up	100.65	91.56	9.15	162.91	77.36	18.10	263.56	239.75	13.18
Sandy Beach	-0.53	-15.32	-0.05	-1.26	-43.00	-0.14	-1.79	-51.73	-0.09
Waterbody	44.04	19.89	4.00	16.64	6.27	1.85	60.69	27.41	3.03
Wetland	-44.65	-11.61	-4.06	-115.87	-34.08	-12.87	-160.52	-41.73	-8.03
Vegetation	87.96	69.75	8.00	-42.43	-19.82	-4.71	45.54	36.11	2.28
Crop land	-53.92	-45.00	-4.90	20.44	31.02	2.27	-33.47	-27.94	-1.67
Barren land	-27.75	-80.95	-2.52	1.26	19.30	0.14	-26.49	-77.28	-1.32
Fallow land	-29.25	-56.87	-2.66	132.94	599.10	14.77	103.68	201.52	5.18
Plantation	-76.56	-11.80	-6.96	-175.35	-30.64	-19.48	-251.91	-38.82	-12.60

*In LULC features, negative values signify area loss, whereas positive values indicate area gain.

The percentage and rate of change were calculated using Eq. (4) and (5), respectively.

Built-up area shows a steep and consistent increase, expanding by + 100.65 km^2^ (91.56%) during 2004–2015, + 162.91 km^2^ (77.36%) during 2015–2024, and a total of + 263.56 km^2^ (239.75%) between 2004 and 2024, with corresponding rates of change of 9.15, 18.10, and 13.18 km^2^/year, respectively. The high percentage values indicate the relative magnitude of urban expansion, while the rate of change reflects its accelerating pace over time. The study region lies within the coastal parts of Alappuzha and Ernakulam districts, where urbanization has been closely linked to population growth and settlement expansion^6^. According to the 2011 Census, Ernakulam (68.07%) ranked first and Alappuzha (54.00%) ranked fifth in terms of urbanization. This trend is largely driven by the transformation of peripheral rural areas into urban centres and the growth of the tertiary sector^67^. Kochi Corporation emerges as the most urbanized, with its built-up area increasing from 42.86 km^2^ in 2004 to 63.26 km^2^ in 2024, followed by urban centres such as Alappuzha, Mavelikkara, Kayamkulam, Ambalapuzha, Cherthala, Thrikkakara, and North Paravoor. Such expansion of settlements, particularly along the coast, has resulted in large-scale encroachment of coastal land cover, intensifying ecosystem degradation and coastal vulnerability.

Plantation experienced the steepest decline, losing –76.56 km^2^ (–11.80%) during 2004–2015, –175.35 km^2^ (–30.64%) during 2015–2024, and a total of –251.91 km^2^ (–38.82%) over 2004–2024, at rates of –6.96, –19.48, and –12.60 km^2^/year, reflecting extensive conversion along the coast where plantations serve as vital buffers for ecosystems and communities^6^. Vegetation initially increased by + 87.96 km^2^ (69.75%) at 8.00 km^2^/year during 2004–2015 due to the conversion of wetland and plantation areas into green cover, but declined by –42.43 km^2^ (–19.82%) at –4.71 km^2^/year during 2015–2024 owing to urban expansion and further land conversion, resulting in a net gain of + 45.54 km^2^ (36.11%) over 2004–2024 at 2.28 km^2^/year. Vegetation remains largely confined to riverbanks and tidal creeks, consistent with^68^, who reported similar mangrove restrictions in Ernakulam due to land conversion, urbanization, and altered hydrodynamics. Barren land steadily declined by –27.75 km^2^ (–80.95%) during 2004–2015 at –2.52 km^2^/year, slightly recovered by + 1.26 km^2^ (19.30%) at 0.14 km^2^/year during 2015–2024, and overall decreased by –26.49 km^2^ (–77.28%) over 2004–2024 at –1.32 km^2^/year, indicating a long-term reduction of uncultivated and degraded areas.

Waterbody in the study area expanded consistently (+ 60.69 km^2^, + 27.41%) between 2004–2024, indicating improved surface water distribution across rivers, lakes, and tributaries^23^, though temporary crop inundation during the Puncha season likely caused slight overestimation in LULC maps. Wetlands, however, declined sharply (–160.52 km^2^, –41.73%) due to conversion of marshy, aquaculture, and paddy areas such as Kuttanadu, Kole, Pokkali, and Kaipad^68^. Cropland decreased (–33.47 km^2^, –27.94%) as agricultural land was converted to built-up and fallow areas, while fallow land rose markedly (+ 103.68 km^2^, + 201.52%) reflecting shifts in cultivation and land-use practices. Sandy beach areas also contracted slightly (–1.79 km^2^, –51.73%) from 2004–2024, mainly due to coastal erosion, tidal activity, and anthropogenic pressures along Alappuzha, Mararikulam, Chellanam, and nearby stretches.

Table 8 thus shows that between 2004 and 2024, built-up areas and fallow land increased significantly, whereas plantation, wetlands, crop land, barren land, and sandy beaches declined, with vegetation and waterbody recording a modest net gain, reflecting the combined impacts of urbanization, land-use conversion, and coastal processes on the region’s landscape.

### LULC transition dynamics (2004–2024)

**Fig. 10:**
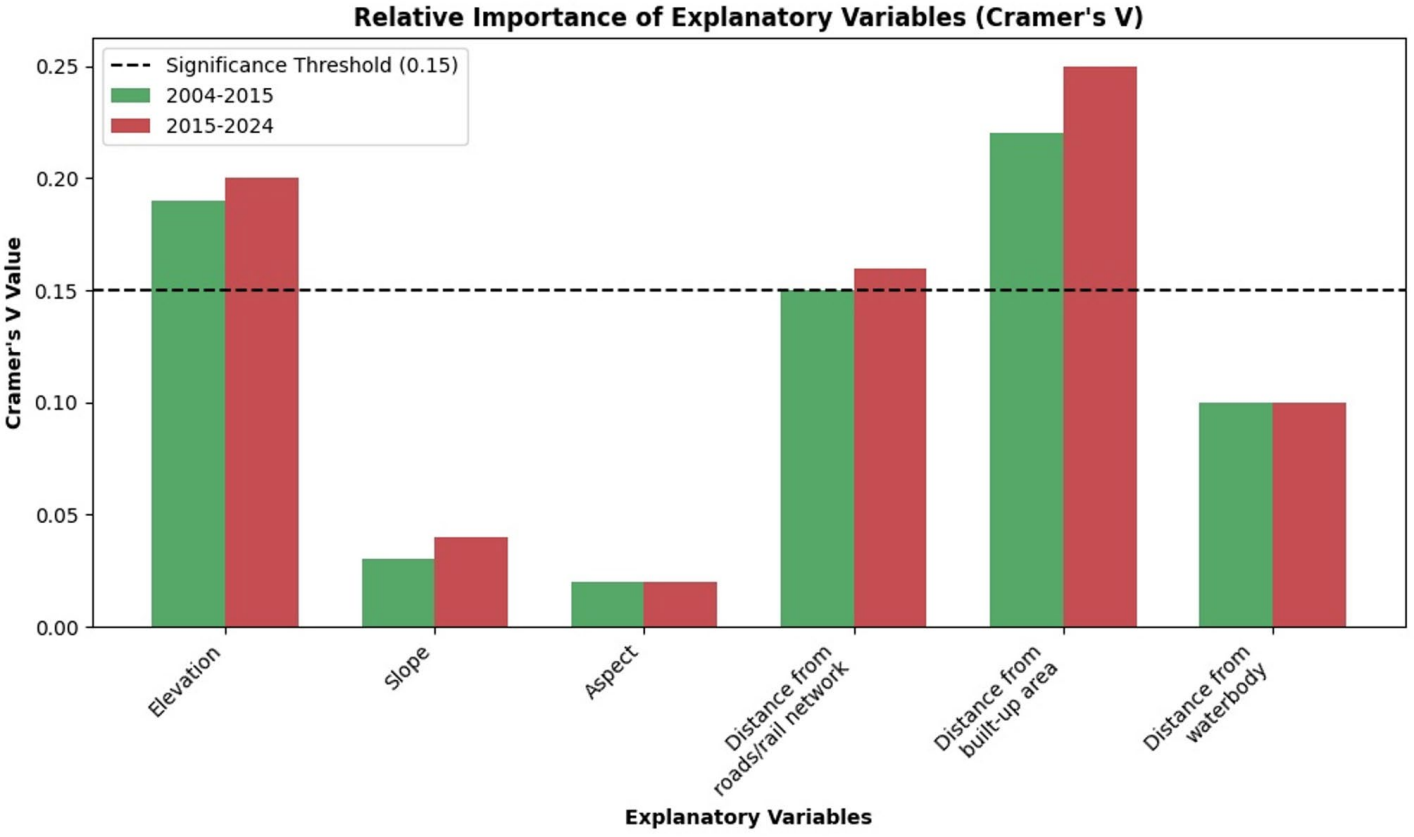
Relative importance of explanatory variables (Cramer’s V).

The Cramer’s V test was performed to identify the association between the driver variables and land cover classes (LULC). The Cramer’s V values were measured between the LULC and driver variables for the intervals 2004–2015 and 2015–2024, as shown in Table 9 and Fig. 10. Figure 10 presents the relative importance of each explanatory variable in driving the LULC transitions based on these values. Cramer’s V values greater than 0.15 were considered significant for inclusion in the model to simulate transition potential maps^62^. From Table 9, it is evident that among the topographical variables, elevation shows the highest association with LULC, with values of 0.19 and 0.20 for the intervals 2004–2015 and 2015–2024, respectively, indicating a moderate influence on land cover changes. The Euclidean distance from built-up area also exhibits relatively strong associations (0.22 and 0.25), highlighting its significant impact on urban expansion. Meanwhile, the Euclidean distance from roads/rail network shows moderate influence (0.15 and 0.16), suggesting that proximity to infrastructure moderately affects land transformation. In contrast, slope, aspect, and distance from waterbody display very low values (< 0.1), implying weak or negligible influence on LULC changes and are therefore less relevant and excluded from the transition potential modelling.

**Table 9:** Cramer’s V values indicating the correlation between LULC changes and explanatory variables for the periods 2004–2015 and 2015–2024.

Explanatory variable	Cramer’s V values	
	2004–2015	2015–2024
Elevation	0.19	0.20
Slope	0.03	0.04
Aspect	0.02	0.02
Euclidean distance from roads/rail network	0.15	0.16
Euclidean distance from built-up area	0.22	0.25
Euclidean distance from waterbody	0.10	0.10

**Fig. 11:**
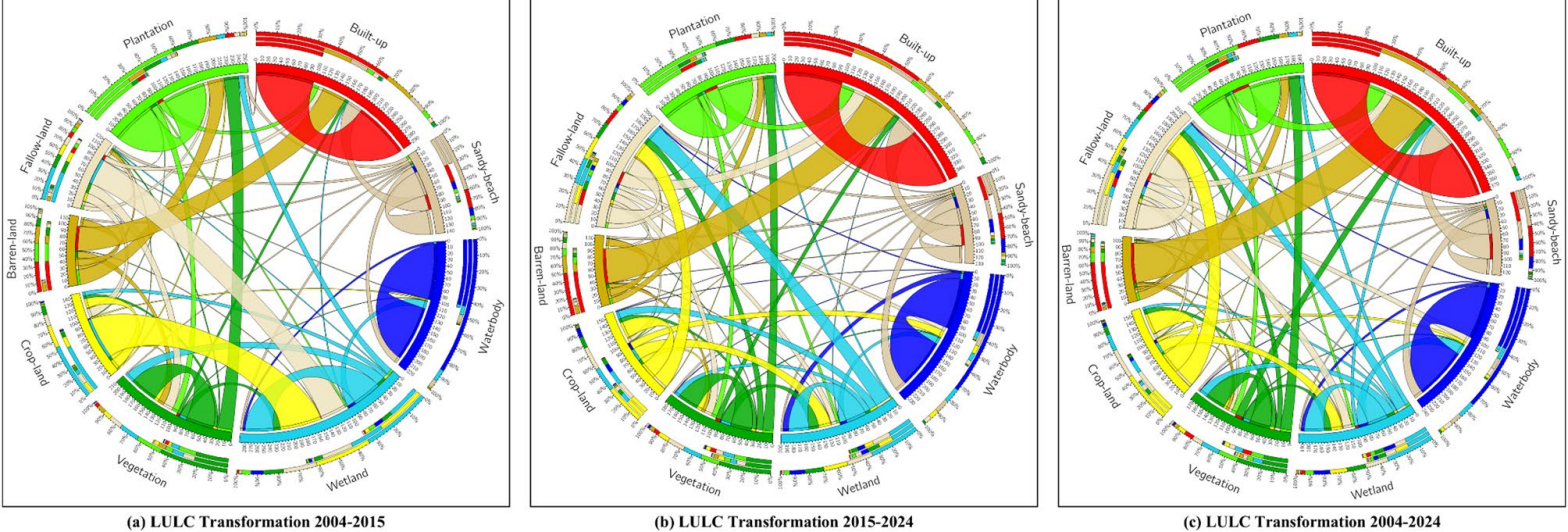
Circus plot showing LULC transitions in the study area for (**a**) 2004–2015, (**b**) 2015–2024, and (**c**) 2004–2024.

Transition potential modelling assesses the likelihood of LULC changes between classes based on area suitability and driving forces, using a transition matrix to estimate the probability of each class converting into another^2^. In TerrSet v.20, the LCM module applied the MLP algorithm to dominant transitions in the study area, achieving accuracy rates exceeding 70% for each sub-model. The resulting transition potential maps served as inputs for the Markov Chain model to predict future changes. The transition matrices for 2004–2015, 2015–2024, and 2004–2024 (Tables 10, 11 and 12) show the projected land cover conversions across the study area. In these matrices, bolded diagonal values represent class persistence, while off-diagonal values indicate transitions between classes. Figure 11 visualizes LULC transformations for 2004–2015 and 2015–2024 using circus plots, highlighting the scale and direction of conversions among land cover types.

**Table 10:** LULC transition matrix showing the area (sq. km) of land cover changes from 2004 – 2015.

	Class Names	Land Area in 2015 (sq.km.)									Area of Transformation (sq.km)
		Built-up area	Sandy Beach	Waterbody	Wetland	Vegetation	Crop land	Barren land	Fallow land	Plantation	
Land Area in 2004 (sq.km.)	Built-up area	107.69	0.046	0.07	0.05	0.43	0.00	0.06	0.10	1.48	2.24
	Sandy Beach	0.91	1.46	0.37	0.15	0.06	0.00	0.12	0.18	0.21	2.00
	Waterbody	0.26	0.49	210.38	9.05	0.36	0.23	0.03	0.42	0.20	11.05
	Wetland	8.59	0.41	49.36	189.23	60.99	27.31	0.28	8.51	40.00	195.45
	Vegetation	6.16	0.21	1.13	15.68	60.84	6.56	0.56	2.66	32.33	65.28
	Crop land	0.18	0.01	2.83	86.21	8.14	18.62	0.02	0.89	0.89	99.17
	Barren land	14.51	0.13	0.03	0.42	3.84	0.56	3.99	2.00	8.78	30.28
	Fallow land	4.06	0.16	0.76	22.92	9.00	7.68	0.26	3.23	3.37	44.85
	Plantation	68.22	0.03	0.53	16.31	70.41	4.92	1.20	4.21	483.10	165.84
	Area contributed to LULC (sq.km)	102.89	1.47	55.09	150.80	153.24	47.27	2.53	18.96	87.26

**Table 11:** LULC transition matrix showing the area (sq. km) of land cover changes from 2015 to 2024.

	Class Names	Land Area in 2024 (sq.km.)									Area of Transformation (sq.km)
		Built-up area	Sandy Beach	Waterbody	Wetland	Vegetation	Crop land	Barren land	Fallow land	Plantation	
Land Area in 2015 (sq.km.)	Built-up area	210.25	0.013	0.02	0.01	0.04	0.00	0.02	0.03	0.10	0.23
	Sandy Beach	1.01	0.82	0.54	0.08	0.10	0.02	0.11	0.15	0.02	2.03
	Waterbody	1.10	0.43	226.88	25.29	3.79	2.44	0.01	4.87	0.50	38.44
	Wetland	4.97	0.11	37.65	117.46	30.39	45.44	0.80	92.50	10.57	222.43
	Vegetation	16.37	0.04	5.06	36.29	77.70	14.79	2.79	23.28	37.71	136.34
	Crop land	0.57	0.00	8.05	13.79	6.04	18.43	0.23	17.99	0.78	47.46
	Barren land	3.83	0.08	0.01	0.06	0.38	0.12	1.17	0.21	0.66	5.35
	Fallow land	4.85	0.04	1.53	2.05	2.97	1.38	1.02	6.29	2.03	15.87
	Plantation	130.55	0.14	2.36	29.12	50.24	3.71	1.64	9.80	344.66	227.56
	Area contributed to LULC (sq.km)	163.24	0.85	55.22	106.70	93.95	67.91	6.62	148.84	52.37

**Table 12:** LULC transition matrix showing the area (sq. km) of land cover changes from 2004 – 2024.

	Class names	Land Area in 2024 (sq.km.)									Area of transformation (sq.km)
		Built-up area	Sandy beach	Waterbody	Wetland	Vegetation	Crop land	Barren land	Fallow land	Plantation	
Land Area in 2004 (sq.km.)	Built-up area	109.33	0.01	0.06	0.01	0.09	0.03	0.01	0.05	0.28	0.54
	Sandy Beach	1.46	0.86	0.53	0.12	0.11	0.01	0.09	0.17	0.04	2.52
	Waterbody	0.76	0.33	200.32	12.69	1.85	0.86	0.06	4.35	0.19	21.10
	Wetland	22.18	0.19	65.48	130.94	54.06	19.76	2.20	64.58	25.09	253.53
	Vegetation	16.61	0.06	2.52	15.38	44.66	9.93	1.43	10.98	24.57	81.46
	Crop land	0.67	0.00	5.93	23.21	5.04	43.28	0.11	40.28	1.29	76.53
	Barren land	23.46	0.09	0.09	0.55	1.79	0.51	1.40	1.44	4.94	32.88
	Fallow land	6.96	0.03	5.42	7.88	3.16	4.11	0.73	22.33	0.85	29.13
	Plantation	192.11	0.09	1.87	33.42	60.96	7.87	1.75	10.98	339.90	309.05
	Area contributed to LULC (sq.km)	264.21	0.80	81.89	93.26	127.06	43.07	6.38	132.81	57.23

Based on Fig. 11a and Table 10, the LULC transition between 2004 and 2015 shows prominent spatial dynamics driven by urban expansion and land conversion. The plantation class showed the highest land conversion, with about 68.22 km^2^ changing into built-up areas and 70.41 km^2^ converting into vegetation. Croplands experienced major losses, particularly transitioning to wetlands (86.21 km^2^) and vegetation (8.14 km^2^). Wetlands, in turn, were highly dynamic, losing significant area to vegetation (60.99 km^2^) and waterbodies (49.36 km^2^) yet also expanding into plantation (40 km^2^) and crop land (27.31 km^2^). The built-up area markedly increased by 102.89 km^2^, predominantly sourced from plantations (68.22 km^2^), barren land (14.51 km^2^), and wetlands (8.59 km^2^), highlighting rapid urbanization. Vegetation showed a dual trend of expansion (153.24 km^2^ gained) and contraction (65.28 km^2^ lost), indicating active land turnover. Meanwhile, while fallow (44.85 km^2^) and barren lands (30.28 km^2^) underwent moderate transitions.

Based on Fig. 11b and Table 11, the LULC transformation between 2015 and 2024 reveals ongoing landscape dynamics, particularly driven by urbanization and ecological shifts. Built-up areas continued expanding significantly, gaining 163.24 km^2^, primarily from plantation (130.55 km^2^) and vegetation (16.37 km^2^). Wetlands again played a dynamic role, losing 222.43 km^2^ of their area but contributing to waterbodies (37.65 km^2^), vegetation (30.39 km^2^), and especially fallow land (92.50 km^2^), indicating changing land-use conditions in the coastal region. Plantation areas saw major reductions, contributing over 52 km^2^ to other classes, with substantial conversion to built-up (130.55 km^2^), wetlands (29.12 km^2^), and vegetation (50.24 km^2^). Vegetation remained transitional, gaining from wetland (30.39 km^2^) and plantation (50.24 km^2^) while losing to built-up (16.37 km^2^), fallow lands (23.28 km^2^), wetland (36.29 km^2^), and plantation (37.71 km^2^). Crop land declined in stability, contributing 27.63 km^2^ to other uses, particularly to wetland (13.79 km^2^) and vegetation (6.04 km^2^), while gaining from wetland (45.44 km^2^), highlighting agricultural land redistribution. Fallow land increased by 148.84 km^2^, mainly from wetland (92.50 km^2^), vegetation (23.28 km^2^), and crop land (17.99 km^2^). Barren land remained relatively stable by gaining 6.62 km^2^ and contributing a modest 5.35 km^2^ to other classes. Waterbodies slightly expanded, gaining 55.22 km^2^, largely from wetland (37.65 km^2^) and cropland (8.05 km^2^). Sandy beach remained the least dynamic class, with minimal changes (only 0.85 km^2^ gained).

Between 2004 and 2024, the LULC transformation (as shown in Fig. 11c and Table 12) highlights extensive land cover changes across all classes over the two-decade period. Built-up areas exhibited the most substantial growth, gaining 264.21 km^2^, primarily from plantation (192.11 km^2^), wetland (22.18 km^2^), vegetation (16.61 km^2^), and barren land (23.46 km^2^), clearly indicating persistent urban expansion. Plantation areas, one of the largest land sources of change, contributed 22.56 km^2^ to other classes, particularly to built-up, wetland (33.42 km^2^), and vegetation (60.96 km^2^), while retaining a core area of 339.90 km^2^. Wetlands were highly dynamic, contributing 253.53 km^2^ to other classes, especially to built-up (22.18 km^2^), vegetation (65.48 km^2^), and notably fallow land (64.58 km^2^), and gaining land from cropland (23.21 km^2^) and plantation (33.42 km^2^). Vegetation saw mixed transitions, losing to built-up (16.61 km^2^), wetland (15.38 km^2^), and plantation (24.57 km^2^), but also gaining 127.06 km^2^ from classes such as wetland (54.06 km^2^) and plantation (60.96 km^2^). Cropland showed reduced stability, with 76.53 km^2^ to other land-use classes, mainly wetland (23.21 km^2^) and fallow land (40.28 km^2^), while also receiving minor gains from vegetation and plantation. Fallow land expanded notably, gaining 132.81 km^2^, mainly from wetlands, cropland, and vegetation. Waterbodies gained 81.89 km^2^, especially from wetlands (65.48 km^2^), pointing to the conversion or inundation of marshy areas. Barren land remained relatively stable with modest transformations, gaining only 6.38 km^2^, but losing 32.88 km^2^ area to built-up and plantation. The sandy beach showed minimal changes throughout the period, with a total gain of just 0.80 km^2^, indicating a static spatial extent. Overall, the 2004–2024 period shows increasing urbanization, reduction in plantation areas, changes in wetlands, and shifting agricultural land-use patterns.

The findings of this study align with previous research conducted in Cochin^19^, Alappuzha district^22,23^, and Ernakulam district^26^, all of which reported a consistent increase in urban areas. This expansion has predominantly occurred at the expense of vegetation, plantations, and barren lands, reflecting widespread land conversion associated with urban growth. Similarly^2^, stressed the need for effective urban planning to manage rapid built-up expansion amid limited land and water resources.

### LULC prediction and validation for 2024

**Fig. 12:**
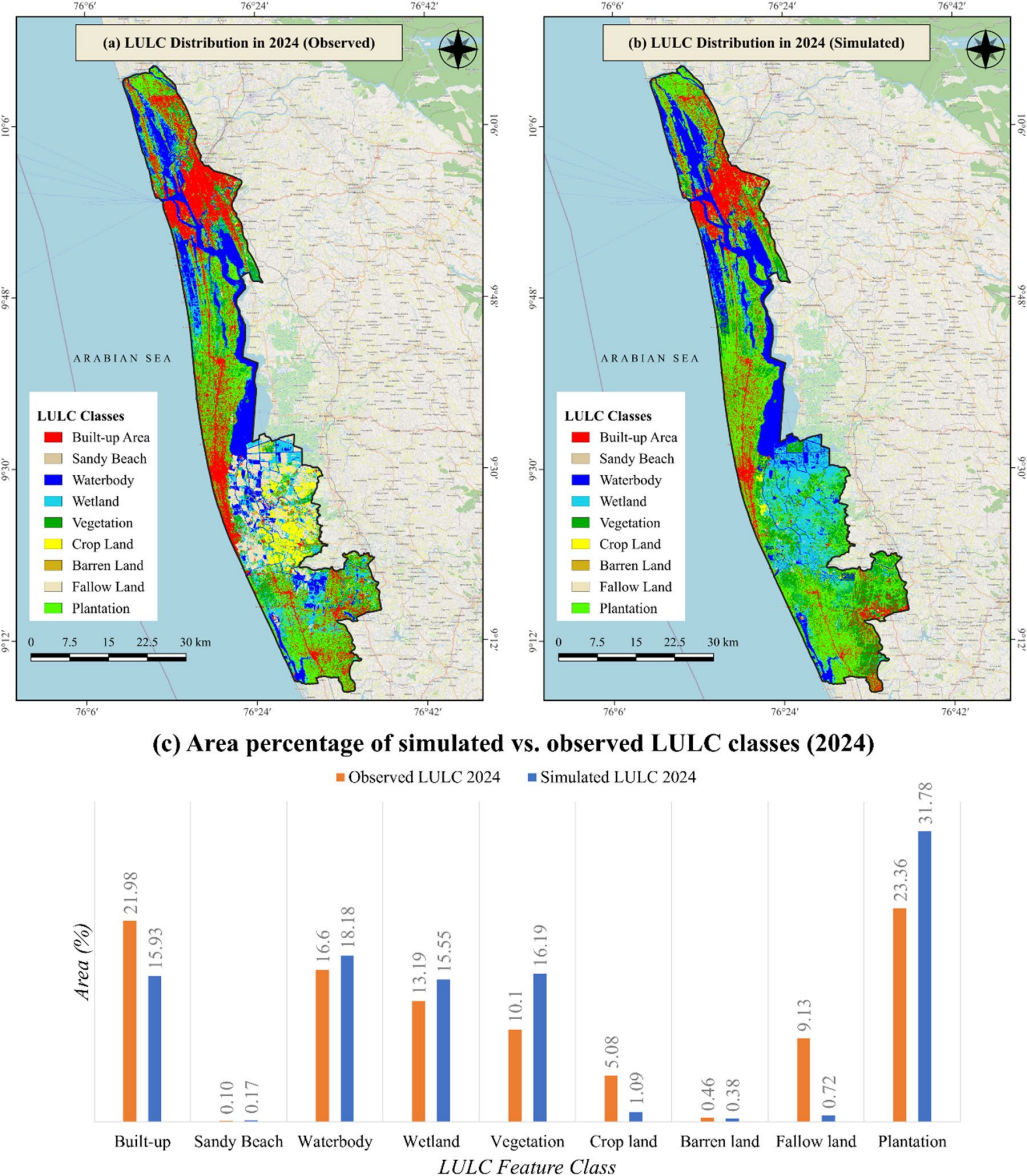
Coastal LULC distribution in 2024 (**a**) RF-based classified LULC, (**b**) Simulated using MLP–Markov Chain model, and (**c**) Comparison of areal statistics of LULC features from observed and simulated maps for the year 2024 (Map Prepared in QGIS 3.44—https://qgis.org/).

Using the categorical LULC maps of 2004 and 2015, the 2024 LULC was predicted employing the MLP–Markov Chain model. Figure 12 presents the projected LULC 2024 map alongside the actual classified 2024 map, with Table 13 highlighting the notable differences between the predicted and observed LULC classes. Built-up areas were significantly underestimated (270.75 km^2^ projected vs. 373.49 km^2^ actual), while plantation and vegetation (540.34 km^2^ and 275.22 km^2^) were overpredicted. Wetlands and waterbodies (264.43 km^2^ and 309.10 km^2^) were slightly overestimated, cropland (18.48 km^2^ projected vs. 86.34 km^2^ actual), and fallow land (12.31 km^2^ projected vs. 155.13 km^2^ actual) were underpredicted. These discrepancies can be attributed to differences in the input datasets, as the LULC maps for 2004 and 2015 were acquired during different months (December and January), representing different stages of the cultivation cycle.

**Table 13:** Comparison of simulated and observed LULC distribution for 2024.

Land use and land cover feature	Simulated 2024		Observed 2024	
	Area (sq. km.)	Percentage (%)	Area (sq. km.)	Percentage (%)
Built-up	270.75	15.93	373.49	21.98
Sandy Beach	2.93	0.17	1.67	0.10
Waterbody	309.10	18.18	282.11	16.60
Wetland	264.43	15.55	224.16	13.19
Vegetation	275.22	16.19	171.66	10.10
Crop land	18.48	1.09	86.34	5.08
Barren land	6.53	0.38	7.79	0.46
Fallow land	12.31	0.72	155.13	9.13
Plantation	540.34	31.78	397.03	23.36

To assess the model’s performance, a comparison was made between the simulated and actual LULC map of 2024, with validation carried out through the VALIDATE module in TerrSet v.20. The model is considered valid as the Kstandard (overall Kappa) score of 0.7181 slightly exceeds the 70% threshold, indicating moderate overall agreement between the projected and actual LULC maps. However, certain LULC classes, particularly built-up areas, cropland, fallow land, vegetation, and plantations, showed noticeable differences between the simulated and observed areas due to the dynamic and heterogeneous nature of coastal land-use systems. Nevertheless, the other Kappa indices, Kno (0.8535), Klocation (0.8079), and KlocationStrata (0.8079), all exceed 80%, demonstrating strong agreement in terms of quantity and spatial accuracy. This indicates that the model was able to reasonably capture the overall spatial distribution and dominant LULC transition trends across the study area, supporting its applicability for regional-scale predictive analysis.

The statistical measures, AgreementChance = 0.1000 (10.00%), AgreementQuantity = 0.4322 (43.22%), AgreementGridCell = 0.3359 (33.59%), DisagreementGridCell = 0.0799 (7.99%), and DisagreementQuantity = 0.0520 (5.20%), indicate the level of agreement between the simulated and reference maps. In this case, overall disagreement is low, with quantity errors (5.20%) slightly lower than allocation errors (7.99%), indicating that the model predicts LULC class proportions more accurately than their exact spatial locations. The overall agreement of 86.81% demonstrates that the model effectively reproduces both the distribution and proportions of LULC classes, showing good predictive performance with minimal error.

### Projection of future LULC scenarios (2034, 2044, and 2054)

**Fig. 13:**
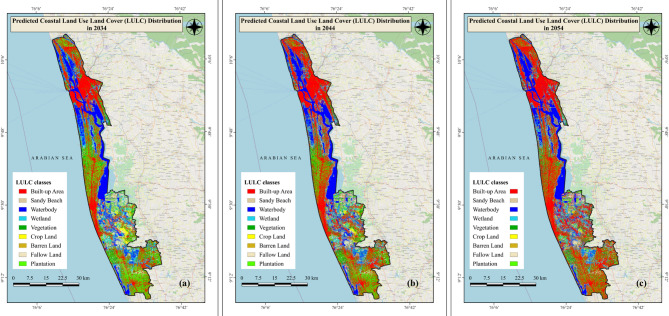
Predicted coastal LULC distribution for the years (**a**) 2034, (**b**) 2044, and (**c**) 2054.

**Fig. 14:**
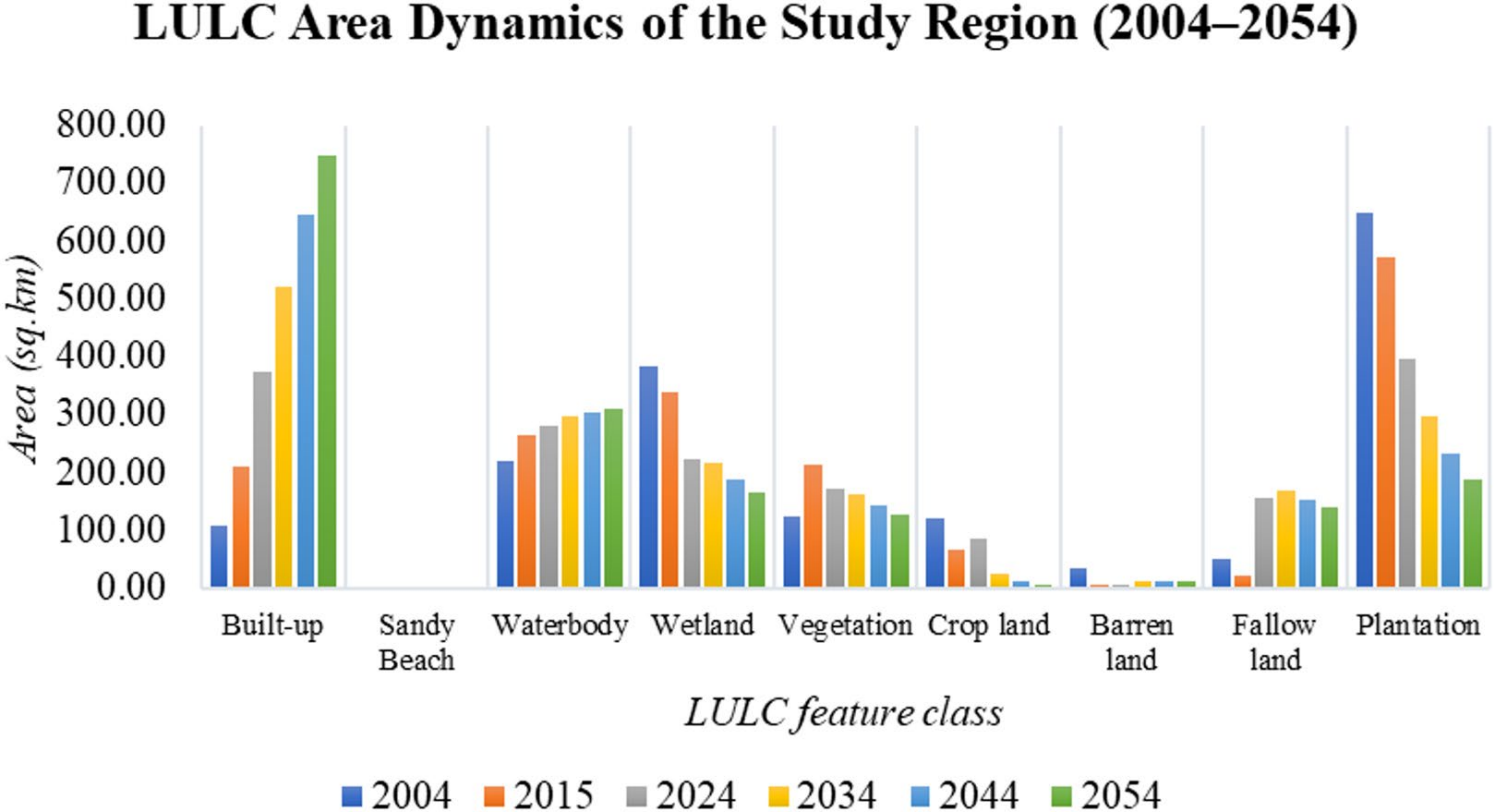
Observed (2004–2024) and predicted (2024–2054) area distribution of LULC classes from 2004 to 2054, showing temporal trends in land use dynamics.

The future Land Use and Land Cover (LULC) changes were predicted for the years 2034, 2044, and 2054 using the Land Change Modeler (LCM). The spatial distribution of the simulated LULC scenarios is illustrated in Fig. 13, while the areal statistics of the future projections are presented in Fig. 14 and Table 14. In LCM, the quantity of change and the spatial allocation of change are determined by the Markov chain and the Multilayer Perceptron (MLP) neural network, respectively. To develop the transition potential maps, major LULC transitions between t₁ (2004), t₂ (2015), and t₃ (2024) were analysed, which formed the basis for defining transition classes. The explanatory variables influencing these transitions were selected using Cramer’s V test, ensuring that only the most relevant driving factors were included. One of the key features of LCM is its ability to utilise MLP–ANN techniques to generate sub-LULC models for change prediction and transition potential mapping. In this study, variables such as elevation, distance from road/rail networks, and distance from built-up areas were used as independent inputs, while the historical land use/cover maps served as dependent inputs. The MLP model was executed with a momentum factor of 0.5 and 10,000 iterations to optimise prediction accuracy.

**Table 14:** Area statistics of different LULC features during 2024–2054.

Land use and land cover feature	2024		2034		2044		2054	
	Area (sq. km.)	Percentage (%)	Area (sq. km.)	Percentage (%)	Area (sq. km.)	Percentage (%)	Area (sq. km.)	Percentage (%)
Built-up	373.49	21.98	521.07	30.66	646.51	38.04	747.52	43.99
Sandy Beach	1.67	0.10	1.53	0.09	1.56	0.09	1.52	0.09
Waterbody	282.11	16.60	296.00	17.42	304.75	17.93	309.15	18.19
Wetland	224.16	13.19	216.23	12.72	188.93	11.12	166.32	9.79
Vegetation	171.66	10.10	162.39	9.56	143.90	8.47	126.79	7.46
Crop land	86.34	5.08	24.15	1.42	11.29	0.66	5.28	0.31
Barren land	7.79	0.46	12.97	0.76	13.39	0.79	12.80	0.75
Fallow land	155.13	9.13	169.34	9.96	154.62	9.10	141.97	8.35
Plantation	397.03	23.36	295.68	17.40	234.41	13.79	188.00	11.06

Based on the projected LULC data and change analysis (Table 15), the coastal stretches of Ernakulam–Alappuzha are expected to undergo significant land cover transformations between 2024 and 2054. Built-up area will be the predominant LULC type. Built-up areas are projected to expand dramatically from 373.49 km^2^ (21.98%) in 2024 to 747.52 km^2^ (43.99%) by 2054, with the highest rate of increase observed between 2024 and 2034 (147.58 km^2^, 39.51%, 14.76 km^2^/year), followed by steady growth in subsequent decades. This expansion likely reflects the continuing urban growth pattern across the coastal stretch, particularly influenced by the rapid urbanisation and infrastructure concentration around Kochi and Ernakulam district^69^. This growth mainly occurs through the conversion of plantation, fallow land, vegetation, and wetland areas, while natural land cover types are projected to decrease over time. Vegetation is expected to decrease from 171.66 km^2^ (10.10%) to 126.79 km^2^ (7.46%), with the most substantial loss occurring between 2034 and 2044 at a rate of 1.85 km^2^/year. Cropland faces the steepest reduction, shrinking from 86.34 km^2^ (5.08%) to 5.28 km^2^ (0.31%) over the 30-year period, indicating rapid conversion to built-up areas. The highest declination is between 2024 and 2034 (-62.19 km^2^, -72.03%, -6.22 km^2^/year), followed by a decrease in subsequent years. Plantation areas are also projected to decline markedly from 397.03 km^2^ (23.36%) to 188.00 km^2^ (11.06%), with losses distributed across all decades but particularly pronounced between 2024 and 2034 (-101.35 km^2^, -25.53%, -10.14 km^2^/year). Wetlands are expected to reduce from 224.16 km^2^ (13.19%) to 166.32 km^2^ (9.79%), mainly between 2034 and 2044 (-27.03 km^2^, -0.13%, -2.73 km^2^/year), increasing pressure on coastal ecosystems. Waterbodies are projected to increase moderately from 282.11 km^2^ (16.60%) to 309.15 km^2^ (18.19%), while sandy beaches remain largely stable (0.10%). Fallow land initially rises between 2024 and 2034 (+ 14.21 km^2^, 9.16%) but declines thereafter, resulting in a net loss of -13.16 km^2^ (-0.44 km^2^/year) by 2054. Barren land shows a slight overall gain (+ 5.01 km^2^, 0.64%) between 2024 and 2054. The relatively stable projections for sandy beach and barren land indicate a limitation of the model, as it is less sensitive to transitions occurring in smaller or low-percentage areas within the study region.

**Table 15:** Spatiotemporal changes in LULC area, percentage, and rate of change during 2024–2034, 2034–2044, 2044–2054, and 2024–2054.

Land use and land cover feature	Change in area (2024–2034) *			Change in area (2034–2044) *			Change in area (2044–2054) *			Change in area (2024–2054) *		
	Area (sq. km.)	Percentage of change (%)	Rate of change (sq.km/year)	Area (sq. km.)	Percentage of change (%)	Rate of change (sq.km/year)	Area (sq. km.)	Percentage of change (%)	Rate of change (sq.km/year)	Area (sq. km.)	Percentage of change (%)	Rate of change (sq.km/year)
Built-up	147.58	39.51	14.76	125.44	0.24	12.54	101.01	0.16	10.10	374.03	1.00	12.47
Sandy Beach	-0.14	-8.38	-0.01	0.03	0.02	0.00	-0.04	-0.03	0.00	-0.15	-0.09	-0.01
Waterbody	13.89	4.92	1.39	8.75	0.03	0.88	4.40	0.01	0.44	27.04	0.10	0.90
Wetland	-7.93	-3.54	-0.79	-27.30	-0.13	-2.73	-22.61	-0.12	-2.26	-57.84	-0.26	-1.93
Vegetation	-9.27	-5.40	-0.93	-18.49	-0.11	-1.85	-17.11	-0.12	-1.71	-44.87	-0.26	-1.50
Crop land	-62.19	-72.03	-6.22	-12.86	-0.53	-1.29	-6.01	-0.53	-0.60	-81.06	-0.94	-2.70
Barren land	5.18	66.50	0.52	0.42	0.03	0.04	-0.59	-0.04	-0.06	5.01	0.64	0.17
Fallow land	14.21	9.16	1.42	-14.72	-0.09	-1.47	-12.65	-0.08	-1.27	-13.16	-0.08	-0.44
Plantation	-101.35	-25.53	-10.14	-61.27	-0.21	-6.13	-46.41	-0.20	-4.64	-209.03	-0.53	-6.97

*In LULC features, negative values signify area loss, whereas positive values indicate area gain.

The percentage and rate of change were calculated using Eq. (4) and (5), respectively.

The projections reveal a clear trend of urban expansion at the expense of agricultural, vegetative, wetland, and plantation areas, indicating a shift toward highly anthropogenic land uses. Significant urbanization is observed in Kochi Corporation and municipalities such as North Paravoor, Thripunithura, Kalamassery, Cherthala, Alappuzha, Kayamkulam, and Mavelikkara. In particular, the taluks of Kochi, Kanayannur, Paravoor, and Ambalappuzha are projected to experience substantial urban growth from 2004 to 2054^19^. Similarly identified Cochin and Kanayannur as the most urbanized taluks. Supporting this^22,23^, noted that four of the six municipalities in Alappuzha District (Alappuzha, Cherthala, Kayamkulam, Mavelikkara, Chengannur, and Haripad) located in the southern region, are explaining the increase in population density and built-up land in this area^70^. Further observed that major cities in Kerala, including Kochi, Thiruvananthapuram, and Kozhikode, exhibit urbanization patterns similar to larger Indian cities, characterized by the proliferation of high-rise buildings and the reduction of green and open spaces. Their study also emphasized that the distribution of million-plus cities (e.g., Kochi) and urban agglomerations (Kochi, Cherthala, Alappuzha, and Kayamkulam) in Kerala predominantly occurs in low-lying coastal areas, underscoring the centrality of the coastal zone in the state’s urban development. Additionally^23^, noted that rural-to-urban migration toward district centres and tourism growth around Vembanad Lake have significantly contributed to the expansion of built-up land in the prime municipal areas of Alappuzha District. Moreover, natural land cover types such as cropland, wetland, and plantation have undergone considerable reductions in area. Cropland and plantation areas have particularly contributed to the process of urban infrastructure development^71^. A study by^72^, also reported a reduction in the aerial extent of the Cochin estuarine system by 12.47 km^2^, attributing this to major geomorphic alterations across several segments. Therefore, future land-use activities must be guided by appropriate development and regulatory frameworks to mitigate the long-term adverse impacts of LULC changes.

The analysis of LULC change rates and magnitudes indicates that the most rapid transformations are expected during the first decade (2024–2034), followed by continued but slower conversions in subsequent decades. This aligns with the findings of^19^, who projected major LULC alterations in Cochin city between 2020 and 2045. These trends collectively underscore the urgent need for sustainable land management, spatial planning, and conservation strategies to mitigate the environmental and ecological consequences of rapid urbanization in Kerala’s coastal zone.

### Model limitations and uncertainty

This study utilized GEE for LULC classification of 2004, 2015, and 2024, and employed the Land Change Modeler (LCM) in TerrSet v.20 to predict changes for 2034, 2044, and 2054 across the coastal stretches of Ernakulam–Alappuzha. Although GEE provides an efficient platform for large-scale geospatial analysis, limitations such as restricted machine learning algorithms, constraints on training samples and input features, and cloud contamination in tropical regions may influence classification accuracy^73,74^. In this study, persistent cloud cover prevented the use of the December 2014 Landsat image, requiring substitution with a January 2015 image, which introduced minor inconsistencies due to differences in cultivation patterns. LULC prediction using the LCM is also subject to uncertainties associated with satellite data biases, classification errors, and model assumptions^19,75^. Although the simulated outputs showed good agreement with the reference LULC map, slight overestimations and underestimations of certain classes were observed. Moreover, uncertainties related to classification errors, transition-potential modelling, and the stationarity assumption of the Markov Chain may propagate through long-term simulations, particularly for projections extending to 2054 under changing climate conditions, sea-level rise, and evolving coastal policies^76^. To minimise the influence of data errors and model uncertainties, consistent driving factors, filters, and simulation settings were maintained throughout the modelling process (Wang et al. ^77^). Furthermore, the black-box nature of the MLP-based LCM limits model interpretability and the ability to fully explain the influence of individual driving factors on urban growth patterns^40,78^. Future studies could integrate advanced machine learning approaches such as gradient-boosting algorithms^40^, hybrid intelligence frameworks^79^, and Explainable Artificial Intelligence (XAI) techniques to improve the predictive accuracy, transparency, and interpretability of simulations. Furthermore, while the model incorporated key physical and proximity-based driving factors, additional socio-economic and environmental variables could further improve the accuracy and reliability of future LULC simulations^80^.

## Conclusion

Decadal LULC variations reflect the socio-economic transformations of the region and serve as indicators of vulnerability to both natural and anthropogenic pressures. This study integrated remote sensing (RS), GIS, and LCM prediction models to examine past, present, and future LULC dynamics across the coastal stretch of Ernakulam–Alappuzha districts. Landsat ETM + 7 (2004), OLI 8 (2015), and OLI 9 (2024) datasets were used for classification using RF, SVM, and CART algorithms on the GEE platform, where the RF classifier demonstrated the best performance with overall accuracies of 95.46%, 92.38%, and 93.94%, and corresponding Kappa coefficients of 0.95, 0.91, and 0.93 for 2004, 2015, and 2024, respectively. The consistently high precision, recall, and F1-scores (mostly > 90%) confirm the reliability of RF for accurate LULC mapping and change detection. The integrated analysis of LULC patterns, change, and transition dynamics (2004–2024) reveals extensive land transformation dominated by rapid urbanization and conversion of natural land covers. Built-up areas increased by 239%, from 109.93 km^2^ (6.47%) in 2004 to 373.49 km^2^ (21.98%) in 2024, largely at the expense of plantation (–38.82%), wetland (–41.73%), and cropland (–27.94%). Concurrently, wetlands declined from 22.63% to 13.19%, cropland from 7.05 to 5.08%, barren land from 2.02 to 0.46%, and sandy beaches from 0.20 to 0.10%. While vegetation (7.42% → 10.10%), fallow land (3.03% → 9.13%), and waterbodies (13.02% → 16.60%) showed moderate gains. Transition matrix analysis confirmed plantations, wetlands, and vegetation as the most dynamic classes frequently transitioning into built-up and fallow categories, while built-up land remained the dominant gaining class. Vegetation showed initial regeneration (69.75% during 2004–2015) followed by a decline (–19.82% during 2015–2024), while wetland instability (–41.73%) highlighted growing ecological stress. Urban growth was spatially concentrated around Kochi, Alappuzha, and nearby municipalities, where fallows and croplands were rapidly urbanised around the build-ups and settlements. Using six driving factors, elevation, slope, aspect, distance from roads/railways, built-up areas, and waterbodies, the MLP–Markov Chain (LCM) model effectively simulated future LULC transitions. Validation between the predicted and actual 2024 LULC maps yielded a Kappa index of 0.71, slightly above the 70% threshold, confirming model applicability despite agricultural cycle inconsistencies in the 2015 imagery. The predicted scenarios (2024–2054) indicated a clear dominance of built-up land, which is projected to nearly double from 21.98% in 2024 to 43.99% by 2054, reflecting intensified urban growth. In contrast, natural and semi-natural classes such as plantations, cropland, vegetation, and wetlands collectively decline from about 52% to below 30%, marking a loss of nearly half their spatial extent over three decades. The steepest transformation is expected during 2024–2034, marking the most rapid phase of urbanization. These findings emphasize the urgent need for sustainable land-use planning, ecosystem restoration, and development policies that align with the region’s environmental carrying capacity and the increasing human demand on limited land resources.

## Data Availability

The data that support the findings of this study are available on request from the corresponding author.

## References

[1] Devi, A. R. & Shimrah, T. Modeling LULC using multi-layer perceptron Markov change (MLP-MC) and identifying local drivers of LULC in hilly district of Manipur, India. *Environ. Sci. Pollut. Res.* **30**(26), 68450–68466. 10.1007/s11356-023-27153-4 (2023). 10.1007/s11356-023-27153-4 37126182

[2] Leta, M. K., Demissie, T. A. & Tränckner, J. Modeling and prediction of land use land cover change dynamics based on Land Change Modeler (LCM) in Nashe Watershed, Upper Blue Nile Basin, Ethiopia. *Sustainability* **13**(7), 3740–3740. 10.3390/su13073740 (2021).

[3] Anandkumar, A., Vijith, H., Nagarajan, R., Jonathan, M.P., 2018. Evaluation of decadal shoreline changes in the coastal region of Miri, Sarawak, Malaysia. Coastal Management 95–119. 10.1016/b978-0-12-810473-6.00008-x

[4] Aghajani, H., Sarkari, F. & Fattahi Moghaddam, M. Predicting land use/land cover changes using CA-Markov and LCM models in the metropolitan area of Mashhad, Iran. *Model. Earth Syst. Environ.* **10**(6), 7079–7096. 10.1007/s40808-024-02051-x (2024).

[5] Senthilkumar, C., Alabdulkreem, E., Alruwais, N. & Kavitha, M. Coastal spatial planning using object-based image analysis and image classification techniques. *J. South Am. Earth Sci.* **152**, 105322 (2025).

[6] Kaliraj, S., Chandrasekar, N., Ramachandran, K. K., Srinivas, Y. & Saravanan, S. Coastal landuse and land cover change and transformations of Kanyakumari coast, India using remote sensing and GIS. *Egypt. J. Remote Sens. Space Sci.* **20**(2), 169–185. 10.1016/j.ejrs.2017.04.003 (2017).

[7] Islam, M. R. & Esraz-Ul-Zannat, M. Remote sensing based investigation of coastal LULC dynamics in the coastal region of Bangladesh. *Remote Sens. Appl. Soc. Environ.* **31**, 100982–100982. 10.1016/j.rsase.2023.100982 (2023).

[8] Zaki, A., Buchori, I., Sejati, A. W. & Liu, Y. An object-based image analysis in QGIS for image classification and assessment of coastal spatial planning. *Egypt. J. Remote Sens. Space Sci.* **25**(2), 349–359. 10.1016/j.ejrs.2022.03.002 (2022).

[9] Al Mazroa, A. et al. An analysis of urban sprawl growth and prediction using remote sensing and machine learning techniques. *J. S. Am. Earth Sci.* **142**, 104988–104988. 10.1016/j.jsames.2024.104988 (2024).

[10] Kaliraj, S., Chandrasekar, N. & Magesh, N. S. Evaluation of coastal erosion and accretion processes along the southwest coast of Kanyakumari, Tamil Nadu using geospatial techniques. *Arab. J. Geosci.* **8**(1), 239–253. 10.1007/s12517-013-1216-7 (2015).

[11] Anand, B. et al. Long-term shoreline and LULC change computational analysis in part of the east coast of Tamilnadu using geoinformation tools. *Journal of Sedimentary Environments* **9**(3), 707–726. 10.1007/s43217-024-00191-9 (2024).

[12] Li, J., Chen, Y., Gu, Y., Wang, M. & Zhao, Y. Remote sensing mapping and analysis of spatiotemporal patterns of land use and cover change in the Helong region of the Loess Plateau region (1986–2020). *Remote Sens.* **16**(19), 3738–3738. 10.3390/rs16193738 (2024).

[13] Singh, M. C., Kashyap, D., Sur, K. & Prasad, V. Modeling spatio-temporal land use dynamics in Amritsar district, Punjab, India using machine learning. *Spat. Inf. Res.* **33**(3), 25. 10.1007/s41324-025-00624-1 (2025).

[14] Muhammad, R., Zhang, W., Abbas, Z., Guo, F. & Gwiazdzinski, L. Spatiotemporal change analysis and prediction of future land use and land cover changes using QGIS MOLUSCE plugin and remote sensing big data: A case study of Linyi, China. *Land* **11**(3), 419–419. 10.3390/land11030419 (2022).

[15] Dharmesh, M., Sur, K., Bhandari, S., Zala, L. B. & Verma, V. K. Erratic dynamics of LULC over the temporal window 1978–2017: A case study from western flank of Gulf of Cambay, Gujarat, India. *Safety Extreme Environ.* **3**(3), 203–217. 10.1007/s42797-021-00043-z (2021).

[16] Nath, A. et al. Assessing coastal land-use and land-cover change dynamics using geospatial techniques. *Sustainability* **15**(9), 7398–7398. 10.3390/su15097398 (2023).

[17] Blissag, B., Yebdri, D. & Kessar, C. Spatiotemporal change analysis of LULC using remote sensing and CA-ANN approach in the Hodna basin, NE of Algeria. *Phys. Chem. Earth* **133**, 103535–103535. 10.1016/j.pce.2023.103535 (2023).

[18] Abijith, D. & Saravanan, S. Assessment of land use and land cover change detection and prediction using remote sensing and CA Markov in the northern coastal districts of Tamil Nadu, India. *Environ. Sci. Pollut. Res.* **29**(57), 86055–86067. 10.1007/s11356-021-15782-6 (2021). 10.1007/s11356-021-15782-6 34510357

[19] Devi, A. B., Deka, D., Aneesh, T. D., Srinivas, R. & Nair, A. M. Predictive modelling of land use land cover dynamics for a tropical coastal urban city in Kerala India. *Arabian J. Geosci.* **15**(5), 399. 10.1007/s12517-022-09735-7 (2022).

[20] Dhiman, R., Kalbar, P. & Inamdar, A. B. Spatial planning of coastal urban areas in India: Current practice versus quantitative approach. *Ocean Coast. Manage.* **182**, 104929–104929. 10.1016/j.ocecoaman.2019.104929 (2019).

[21] Tahir, Z. et al. Predicting land use and land cover changes for sustainable land management using CA-Markov modelling and GIS techniques. *Sci. Rep.* **15**(1), 3271. 10.1038/s41598-025-87796-w (2025). 39863826 10.1038/s41598-025-87796-w PMC11762839

[22] Prasad, G. & Ramesh, M. V. Spatio-temporal analysis of land use/land cover changes in an ecologically fragile area—Alappuzha district, southern Kerala, India. *Nat. Resour. Res.* **28**, 31–42. 10.1007/s11053-018-9419-y (2019).

[23] Varunprasath, K., Islam, M.N., Amritha, P.S., 2025. Land use and land cover analysis in the Alappuzha District, South Kerala, India. In: Springer Climate, 291–307. 10.1007/978-3-031-85126-1_11

[24] John, J., Bindu, G. R., Srimuruganandam, B., Wadhwa, A. & Rajan, P. Land use/land cover and land surface temperature analysis in Wayanad district, India, using satellite imagery. *Ann. GIS* **26**(4), 343–360. 10.1080/19475683.2020.1733662 (2020).

[25] Pradeep, A., Ahammed, S. & Rajan, B. P. Analysis of LULC change in Kozhikode Corporation using remote sensing and geographical information system. *J. Geotech. Stud.* **7**(2), 28–35 (2022).

[26] Vilasan, R. T. & Kapse, V. S. Monitoring spatio-temporal dynamics of land use/land cover changes using remote sensing and GIS – A case study of Ernakulam district, India. *Appl. Ecol. Environ. Res.* **20**(4), 3353–3366. 10.15666/aeer/2004_33533366 (2022).

[27] Gupta, A. K., Singh, J. P., Verma, V. K. & Sur, K. Multi-decadal land transformation in South-Western Punjab, India: A case study using geospatial techniques. *Trop. Ecol.* **65**(4), 639–649. 10.1007/s42965-024-00357-6 (2024).

[28] Reddy, K. R., Devaraj, S., Biradar, S., Yarrakula, K. & Srinivas Kumar, K. Spatial distribution of land use/land cover analysis in Hanamkonda taluk, Telangana: A case study Indian. *J. Geo-Marine Sci.* **48**(11), 1761–1768 (2019).

[29] Suresh, D. & Yarrakula, K. InSAR based deformation mapping of earthquake using Sentinel-1A imagery. *Geocarto Int.* **35**(5), 559–568. 10.1080/10106049.2018.1544289 (2020).

[30] Suresh, D. & Yarrakula, K. Subsidence monitoring techniques in coal mining: Indian scenario. *Indian J. Geo-Mar. Sci.* **47**(10), 1918–1933 (2018).

[31] Kaur, H., Tyagi, S., Mehta, M. & Singh, D. Time series (2001/2002–2021) analysis of Earth observation data using Google Earth Engine (GEE) for detecting changes in land use land cover (LULC) with specific reference to forest cover in East Godavari Region, Andhra Pradesh India. *J. Earth Syst. Sci.* **132**(2), 86. 10.1007/s12040-023-02099-w (2023).

[32] Zafar, Z., Zubair, M., Zha, Y., Fahd, S. & Ahmad Nadeem, A. Performance assessment of machine learning algorithms for mapping of land use/land cover using remote sensing data. *Egypt. J. Remote Sens. Space Sci.* **27**(2), 216–226. 10.1016/j.ejrs.2024.03.003 (2024).

[33] Loukika, K. N., Keesara, V. R. & Sridhar, V. Analysis of land use and land cover using machine learning algorithms on Google Earth Engine for Munneru river basin, India. *Sustainability* **13**(24), 13758–13758. 10.3390/su132413758 (2021).

[34] Tesfaye, W., Elias, E., Warkineh, B., Tekalign, M. & Abebe, G. Modeling of land use and land cover changes using Google Earth Engine and machine learning approach: Implications for landscape management. *Environ. Syst. Res.*10.1186/s40068-024-00366-3 (2024).

[35] Bogale, T., Degefa, S., Dalle, G. & Abebe, G. Machine learning-based analysis of land use and land cover trends in southeastern Ethiopia using Google Earth Engine. *Discov. Sustain.*10.1007/s43621-025-01709-5 (2025).

[36] Becker, W. R., Ló, T. B., Johann, J. A. & Mercante, E. Statistical features for land use and land cover classification in Google Earth Engine. *Remote Sensing Applications: Society and Environment* **21**, 100459–100459. 10.1016/j.rsase.2020.100459 (2020).

[37] Khoshnood Motlagh, S. et al. Analysis and prediction of land cover changes using the land change modeler (LCM) in a semiarid river basin, Iran. *Land Degrad. Dev.* **32**(10), 3092–3105. 10.1002/ldr.3969 (2021).

[38] Khan, D. & Khan, N. Decoding urban expansion: A machine learning perspective on Lucknow’s growth trajectory. *GeoJournal*10.1007/s10708-025-11316-6 (2025).

[39] Kim, M., Kim, D. & Kim, G. Examining the relationship between land use/land cover (LULC) and land surface temperature (LST) using explainable artificial intelligence (XAI) models: A case study of Seoul, South Korea. *Int. J. Environ. Res. Public Health* **19**(23), 15926–15926. 10.3390/ijerph192315926 (2022). 36498000 10.3390/ijerph192315926 PMC9740204

[40] Meraj, G. et al. Ensemble machine learning for predicting the urban expansion in Lucknow, India. *Environ. Earth Sci.* **84**(20), 575. 10.1007/s12665-025-12559-9 (2025).

[41] Jain, M. Future land use and land cover simulations with cellular automata-based artificial neural network: A case study over Delhi megacity (India). *Heliyon* **10**(14), e34662–e34662. 10.1016/j.heliyon.2024.e34662 (2024). 39149074 10.1016/j.heliyon.2024.e34662 PMC11324975

[42] Qacami, M., Khattabi, A., Lahssini, S., Rifai, N. & Meliho, M. Land-cover/land-use change dynamics modeling based on land change modeler. *Ann. Reg. Sci.* **70**(1), 237–258. 10.1007/s00168-022-01169-z (2023).

[43] Shafie, B., Javid, A. H., Behbahani, H. I., Darabi, H. & Lotfi, F. H. Modeling land use/cover change based on LCM model for a semi-arid area in the Latian Dam Watershed (Iran). *Environ. Monit. Assess.*10.1007/s10661-022-10876-1 (2023). 36738365 10.1007/s10661-022-10876-1

[44] Ait El Haj, F., Ouadif, L. & Akhssas, A. Simulating and predicting future land-use/land cover trends using CA-Markov and LCM models. *Case Stud. Chem. Environ. Eng.* **7**, 100342–100342. 10.1016/j.cscee.2023.100342 (2023).

[45] Khan, D. & Khan, N. Modelling urban future: Integrating CA-ANN model for comprehensive understanding of land use, land cover changes, and temperature dynamics in Lucknow City, India. *Geol. Ecol. Landsc.* **10**(2), 466–491. 10.1080/24749508.2025.2524207 (2025).

[46] Misra, P., Avtar, R. & Takeuchi, W. Comparison of digital building height models extracted from AW3D, TanDEM-X, ASTER, and SRTM digital surface models over Yangon City. *Remote Sens.* **10**(12), 2008–2008. 10.3390/rs10122008 (2018).

[47] Biney, E. et al. A comprehensive analysis and future projection of land use and land cover dynamics in a fast-growing city: A case study of Sekondi-Takoradi metropolis, Ghana. *Scientific African* **24**, e02207–e02207. 10.1016/j.sciaf.2024.e02207 (2024).

[48] Asare, Y. M., Forkuo, E. K., Forkuor, G. & Thiel, M. Evaluation of gap-filling methods for Landsat 7 ETM+ SLC-off image for LULC classification in a heterogeneous landscape of West Africa. *Int. J. Remote Sens.* **41**(7), 2544–2564. 10.1080/01431161.2019.1693076 (2019).

[49] Gündüz, H. İ. Land-use land-cover dynamics and future projections using GEE, ML, and QGIS-MOLUSCE: A case study in Manisa. *Sustainability* **17**(4), 1363–1363. 10.3390/su17041363 (2025).

[50] Joseph, V. R. Optimal ratio for data splitting. *Stat. Anal. Data Min.* **15**(4), 531–538. 10.1002/sam.11583 (2022).

[51] Zhao, Z. et al. Comparison of three machine learning algorithms using Google Earth Engine for land use land cover classification. *Rangel. Ecol. Manag.* **92**, 129–137. 10.1016/j.rama.2023.10.007 (2024).

[52] Breiman, L., Friedman, J. H., Olshen, R. A. & Stone, C. J. *Classification and Regression Trees* (Routledge, 2017). 10.1201/9781315139470.

[53] Abdelsamie, E. A. et al. Current and potential land use/land cover (LULC) scenarios in dry lands using a CA-Markov simulation model and the classification and regression tree (CART) method: A cloud-based Google Earth Engine (GEE) approach. *Sustainability* **16**(24), 11130–11130. 10.3390/su162411130 (2024).

[54] Abedinia, A. & Seydi, V. Building semi-supervised decision trees with semi-cart algorithm. *Int. J. Mach. Learn. Cybern.* **15**(10), 4493–4510. 10.1007/s13042-024-02161-z (2024).

[55] Raj, A. & Sharma, L. K. Assessment of land-use dynamics of the Aravalli range (India) using integrated geospatial and CART approach. *Earth Sci. Inform.* **15**(1), 497–522. 10.1007/s12145-021-00753-9 (2022).

[56] Breiman, L. Bagging predictors. *Mach. Learn.* **24**(2), 123–140. 10.1007/bf00058655 (1996).

[57] Kumari, A. & Karthikeyan, S. A comparative analysis for forty years of land use land cover change (1991–2021) using CART and random forest classifiers for Varanasi district (India). *SN Comput. Sci.*10.1007/s42979-025-04061-7 (2025).

[58] Ahmad, H. et al. Evaluation and mapping of predicted future land use changes using hybrid models in a coastal area. *Ecol. Inform.* **78**, 102324–102324. 10.1016/j.ecoinf.2023.102324 (2023).

[59] Arya, P. K. et al. Integrating multi-source satellite imagery and socio-economic household data for wealth-based poverty assessment of India: A GIS and machine learning based approach. *Soc. Indic. Res.* **179**(2), 653–676. 10.1007/s11205-025-03614-w (2025).

[60] Sansana, J. et al. Recent trends on hybrid modeling for Industry 4.0. *Comput. Chem. Eng.* **151**, 107365–107365. 10.1016/j.compchemeng.2021.107365 (2021).

[61] Ullah, S. et al. Monitoring effects of LULC change dynamics on the environment using time series remote sensing data with Google Earth Engine. *Theor. Appl. Climatol.* **156**(6), 1–13. 10.1007/s00704-025-05590-0 (2025).

[62] Reddy, C. S. et al. Predictive modelling of the spatial pattern of past and future forest cover changes in India. *J. Earth Syst. Sci.* **126**(1), 1–16. 10.1007/s12040-016-0786-7 (2017).

[63] Sankarrao, L., Ghose, D. K. & Rathinsamy, M. Predicting land-use change: Intercomparison of different hybrid machine learning models. *Environ. Model. Softw.* **145**, 105207–105207. 10.1016/j.envsoft.2021.105207 (2021).

[64] Benhar, H., Idri, A. & Fernández-Alemán, J. L. Data preprocessing for heart disease classification: A systematic literature review. *Comput. Methods Programs Biomed.* **195**, 105635–105635. 10.1016/j.cmpb.2020.105635 (2020). 32652383 10.1016/j.cmpb.2020.105635

[65] Yang, C., Zhai, H., Fu, M., Zheng, Q. & Fan, D. Multi-scenario simulation of land system change in the Guangdong–Hong Kong–Macao Greater Bay Area based on a Cellular Automata–Markov model. *Remote Sens.* **16**(9), 1512–1512. 10.3390/rs16091512 (2024).

[66] Zadbagher, E., Becek, K. & Berberoglu, S. Modeling land use/land cover change using remote sensing and geographic information systems: Case study of the Seyhan Basin, Turkey. *Environ. Monit. Assess.*10.1007/s10661-018-6877-y (2018). 30066225 10.1007/s10661-018-6877-y

[67] Hari, H. U. Urbanization in Kerala: Trends and consequences. *Indian J. Appl. Res.* **5**, 197–200 (2015).

[68] Zainulabdeen, Y. P. & Nagaraj, H. Anthropogenic impacts on wetlands of Kerala, India: A review of literature. *J. Geograph., Environ. Earth Sci. Int.* **26**(6), 28–38. 10.9734/jgeesi/2022/v26i630355 (2022).

[69] Lekshmi, A. & Lancelet, P. T. Trend of urbanisation in Ernakulam with respect to Kerala. *J. Global Resources* **5**, 1–7 (2019).

[70] Krishna, N. G. et al. Understanding the spatio-temporal variation of urbanisation in Kerala, India. *GeoJournal*10.1007/s10708-024-11136-0 (2024).

[71] Krishnan, S. V. & Firoz, M. C. Impact of land use and land cover change on the environmental quality of a region: A case of Ernakulam district in Kerala India. *Reg. Stat.* **11**(2), 102–135. 10.15196/rs110205 (2021).

[72] Kumar, P. K. D., Gopinath, G., Murali, R. M. & Muraleedharan, K. R. Geospatial analysis of long-term morphological changes in Cochin Estuary, SW coast of India. *J. Coast. Res.* **298**, 1315–1320. 10.2112/jcoastres-d-12-00244.1 (2014).

[73] Amani, M. et al. Google Earth Engine cloud computing platform for remote sensing big data applications: A comprehensive review. *IEEE J. Sel. Top. Appl. Earth Obs. Remote Sens.* **13**, 5326–5350. 10.1109/jstars.2020.3021052 (2020).

[74] Zhao, Q. et al. Progress and trends in the application of Google Earth and Google Earth Engine. *Remote Sens.* **13**(18), 3778–3778. 10.3390/rs13183778 (2021).

[75] Lu, Y., Wu, P., Ma, X. & Li, X. Detection and prediction of land use/land cover change using spatiotemporal data fusion and the Cellular Automata–Markov model. *Environ. Monit. Assess.* **191**(2), 1–19. 10.1007/s10661-019-7200-2 (2019). 10.1007/s10661-019-7200-2 30644019

[76] DelSole, T. A fundamental limitation of Markov models. *J. Atmos. Sci.* **57**(13), 2158–2168. 10.1175/1520-0469(2000)057 (2000).

[77] Wang, S. & Zheng, X. Dominant transition probability: Combining CA-Markov model to simulate land use change. *Environ. Dev. Sustain.* **25**(7), 6829–6847. 10.1007/s10668-022-02337-z (2022).

[78] Koutra, S. & Ioakimidis, C. S. Unveiling the potential of machine learning applications in urban planning challenges. *Land* **12**(1), 83–83. 10.3390/land12010083 (2022).

[79] Khan, D., Khan, N. & Ullah, S. Harnessing hybrid intelligence and explainable AI for urban growth prediction: A data-driven framework for sustainable cities. *Environ. Dev. Sustain.*10.1007/s10668-025-06860-7 (2025).

[80] Kale, M. P. et al. Land-use and land-cover change in Western Ghats of India. *Environ. Monit. Assess.* **188**(7), 387. 10.1007/s10661-016-5369-1 (2016). 27256392 10.1007/s10661-016-5369-1

[81] Tucker, C. J. Red and photographic infrared linear combinations for monitoring vegetation. *Remote Sens. Environ.* **8**(2), 127–150. 10.1016/0034-4257(79)90013-0 (1979).

[82] Huete, A. R. A soil-adjusted vegetation index (SAVI). *Remote Sens. Environ.* **25**(3), 295–309. 10.1016/0034-4257(88)90106-x (1988).

[83] Xu, H. A study on information extraction of water body with the modified normalized difference water index (MNDWI). *J. Remote Sens.* **9**(5), 595–600. 10.11834/jrs.20050586 (2005).

[84] Zha, Y., Gao, J. & Ni, S. Use of normalized difference built-up index in automatically mapping urban areas from TM imagery. *Int. J. Remote Sens.* **24**(3), 583–594. 10.1080/01431160304987 (2003).

[85] Zhao, H. & Chen, X. Use of normalized difference bareness index in quickly mapping bare areas from TM/ETM+. *Int. Geosci. Remote Sens. Sympos. (IGARSS)* **3**, 1666–1668. 10.1109/igarss.2005.1526319 (2005).

[86] Rikimaru, A., Roy, P. S. & Miyatake, S. Tropical forest cover density mapping. *Trop. Ecol.* **43**(1), 39–47 (2002).

